# Topological atlas of the hypothalamus in adult rhesus monkey

**DOI:** 10.1007/s00429-020-02093-8

**Published:** 2020-06-16

**Authors:** Anne Marie Wells, Miguel Ángel García-Cabezas, Helen Barbas

**Affiliations:** 1 Graduate Medical Sciences, Boston University School of Medicine, Boston, MA 02215 USA; 2 Department of Health Sciences, Neural Systems Laboratory, Boston University, Boston, MA 02215 USA; 3 Department of Anatomy and Neurobiology, Boston University School of Medicine, Boston, MA USA

**Keywords:** Prosomeric model, Prosomere, Alar plate, Basal plate, Secondary prosencephalon, Prethalamus, Holoprosencephaly

## Abstract

The prosomeric model explains the embryological development of the central nervous system (CNS) shared by all vertebrates as a Bauplan. As a primary event, the early neural plate is patterned by intersecting longitudinal plates and transverse segments, forming a mosaic of progenitor units. The hypothalamus is specified by three prosomeres (hp1, hp2, and the acroterminal domain) of the secondary prosencephalon with corresponding alar and basal plate parts, which develop apart from the diencephalon. Mounting evidence suggests that progenitor units within alar and basal plate parts of hp1 and hp2 give rise to distinct hypothalamic nuclei, which preserve their relative invariant positioning (topology) in the adult brain. Nonetheless, the principles of the prosomeric model have not been applied so far to the hypothalamus of adult primates. We parcellated hypothalamic nuclei in adult rhesus monkeys (*Macaca mulatta*) using various stains to view architectonic boundaries. We then analyzed the topological relations of hypothalamic nuclei and adjacent hypothalamic landmarks with homology across rodent and primate species to trace the origin of adult hypothalamic nuclei to the alar or basal plate components of hp1 and hp2. We generated a novel atlas of the hypothalamus of the adult rhesus monkey with developmental ontologies for each hypothalamic nucleus. The result is a systematic reinterpretation of the adult hypothalamus whose prosomeric ontology can be used to study relationships between the hypothalamus and other regions of the CNS. Further, our atlas may serve as a tool to predict causal patterns in physiological and pathological pathways involving the hypothalamus.

## Introduction

The hypothalamus is a subcortical brain region that lacks consensus on its precise limits and the nuclei contained therein. An understanding of the developmental origin of the hypothalamus can provide a basis to define which limits and nuclei are ontologically hypothalamic, and which are not. In recent decades, two conflicting models have proposed a divergent view on the hypothalamus and adjacent brain regions. The classical columnar model (Herrick 1910) conceptualizes the diencephalon as the sum of four dorsoventral (longitudinal) columns intercalated between the telencephalon (rostrally) and the mesencephalon (caudally). These columns are represented by the epithalamus (top), the dorsal and ventral thalamus (middle), and the hypothalamus (bottom). Thus, the columnar model defines the telencephalon as a different vesicle from the diencephalon and places it as the most rostral part of the neural tube (Herrick 1910; Kuhlenbeck 1973; Swanson 1992, 2003).

A different framework—the prosomeric model—has been proposed to explain the embryological development and evolution of the central nervous system (CNS). Box 1 provides a summary of terms used here to relate the key principles of the prosomeric model. This model is based on genoarchitectonic studies in embryos of vertebrate species and provides a causal basis for the naming of developmental components of the entire CNS (Rubenstein et al. 1994; Puelles and Rubenstein 2003, 2015; Puelles et al. 2012; Nieuwenhuys and Puelles 2016; Puelles 2018). According to the prosomeric model, the progressive regionalization of the CNS begins when the early neural plate is patterned by intersecting longitudinal zones, called plates, and transverse segments, called *protosegments* [secondary prosencephalon (SPro), diencephalon (Dien), mesencephalon (Mes), rhombencephalon (Rhomb)]. These protosegments are further divided into neuromeres with specific names (i.e., the prosomeres of the secondary prosencephalon, mesomeres, rhombomeres). Differential gene expression induced by organizers, which act on the early neuroepithelium along dorsoventral (DV) and anteroposterior (AP) dimensions, specifies a mosaic of *progenitor units* with characteristic molecular profiles. Specifically, neuromeres are the definitive AP subdivisions of the neuroepithelium, sharing a fundamental DV organization into roof, alar, basal, and floor plates, irrespective of their differential AP properties. After specification (primary event), these units expand radially due to histogenetic processes (secondary events) like neurogenesis, neuron migration, and neuron differentiation. The expansion of units across the neural tube varies by the duration and intensity of histogenesis, leading to differential morphogenesis (tertiary event) of CNS structures. The prosomeric model proposes a Bauplan for the development of the neural tube that is identifiable across vertebrates, which can predict homologies of CNS regions that share the same unit of origin in the neural plate and the neural tube (i.e., topological sameness) (Puelles and Ferran 2012; Puelles 2013; Nieuwenhuys and Puelles 2016).

**Fig. 1:**
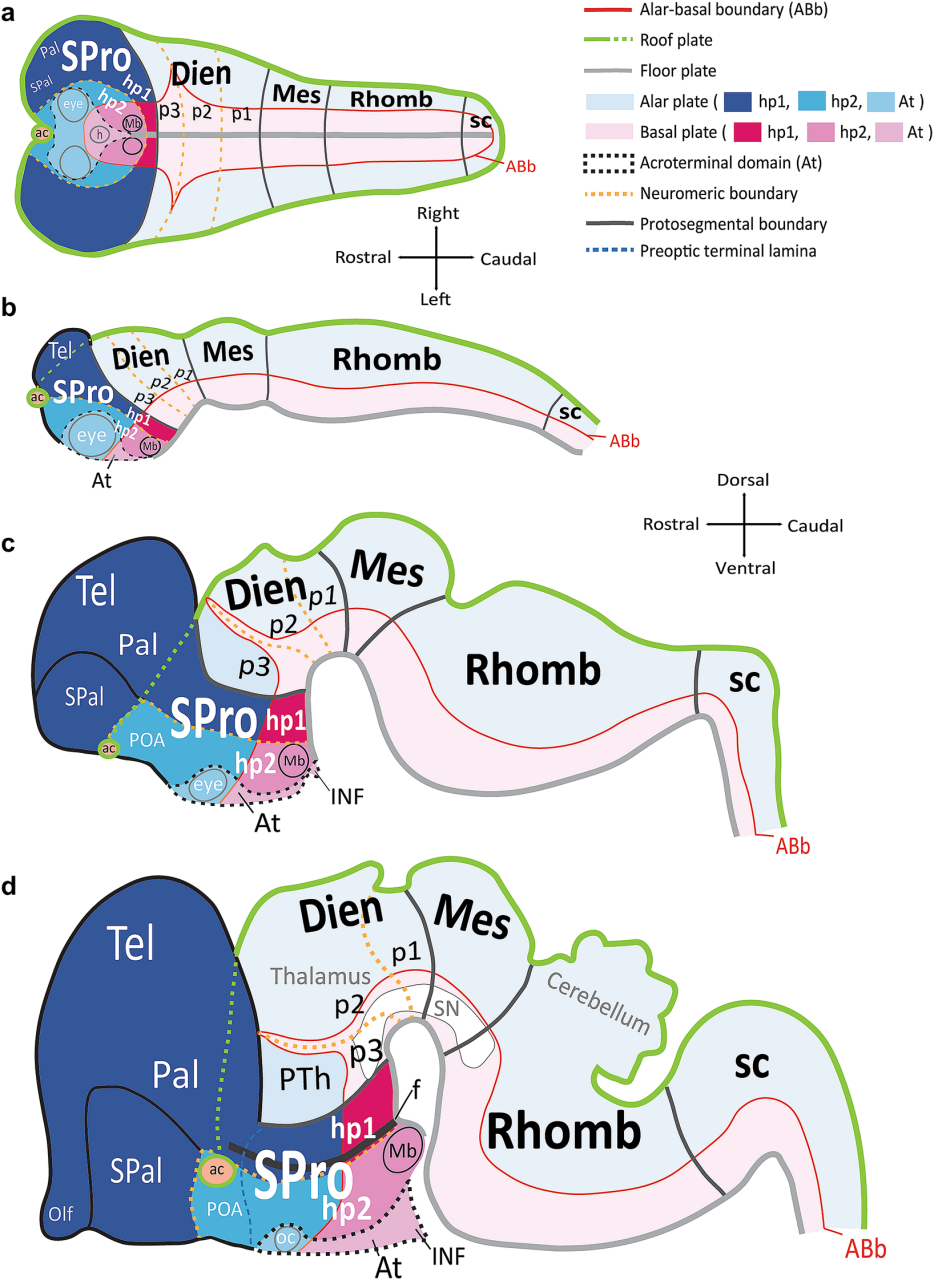
Schematic of progressive regionalization of the hypothalamus according to the prosomeric model. **a** Dorsal view of the neural plate with intersecting longitudinal (rostro-caudal) zones, named plates, and transverse (medio-lateral) segments named protosegments and neuromeres, forming a mosaic of units with unique molecular profiles. The floor plate and the roof plate are organizers whose signals pattern and specify the alar and basal plates. The roof plate encircles the entire neural plate, and the floor plate terminates rostrally just caudal to the prospective mamillary bodies (Mb). Other organizers, like the acroterminal domain (At), pattern and specify transverse segments. The alar-basal boundary (ABb) divides each neuromere into alar plate and basal plate parts. The diencephalic protosegment (Dien; p3, p2, p1), from which the thalamus arises, lies topologically caudal to the neuromeres of the secondary prosencephalon (SPro: hp1, hp2, At). **b** Lateral view from the left of the neural tube shows progressive morphogenesis following neural plate specification into a mosaic of units. Hp1 gives rise to the telencephalon (Tel), including the pallium (Pal) and subpallium (SPal), while hp2 contributes the preoptic area (POA) to the SPal. **c** Lateral view from the left of the neural tube shows progressive morphogenesis and tertiary folding events pushing the hypothalamus to a topographical position ventral to the thalamus, as its name indicates. **d** Lateral view from the left of the neural tube shows progressive morphological expansion of brain regions by neuromere and plate of origin. Several architectonic landmarks, like the fornix, f, the optic chiasm (oc), and the anterior commissure (ac), in the adult brain are visualized as outgrowths along protosegmental and neuromeric boundaries. The sketches of this figure were adapted from Puelles et al. (2008, 2012) and Puelles and Rubenstein (2015). Color code applies to all subsequent figures. See list of abbreviations for complete terms

One of the most significant changes in our understanding of the CNS brought forth by the prosomeric model is the reassignment of the hypothalamus apart from the diencephalon (Fig. 1). The prosomeric model defines five transverse protosegments. These include, from caudal to rostral: the spinal cord (sc), the rhombencephalon (Rhomb), the mesencephalon (Mes), the diencephalon (Dien), and the secondary prosencephalon (SPro). Modern genoarchitectonic studies show that the hypothalamus develops from the three rostral-most *prosomeres* [hp1, hp2, and the acroterminal domain (At)] in the *secondary prosencephalon*, a different protosegment than the diencephalon. Of note, the prosomere that gives rise to the dorsal thalamus (p2) is further separated from the hypothalamus by a prethalamic prosomere (p3) of the diencephalon, which gives rise to the reticular nucleus (R), the zona incerta, and the prethalamic nuclei (classic ventral thalamus).

The early specified transverse limits of p3 that separate the thalamus from the hypothalamus seem to function like other limits in maintaining a relative invariant positioning, or topology, (Nieuwenhuys and Puelles 2016) into adulthood. Another limit, the alar-basal boundary (ABb), subdivides all neuromeres into longitudinal alar plate and basal plate parts. These plates are further subdivided dorsoventrally into smaller (microzonal) progenitor areas, whose respective neurogenic outcome results in hypothalamic nuclei. As such, each adult hypothalamic nucleus can be traced to progenitor areas of either alar or basal plate origin within hp1, hp2, or the acroterminal domain (At) (Puelles et al. 2008, 2012; Ferran et al. 2015; Puelles and Rubenstein 2015). According to the prosomeric model, the telencephalon forms as a vesicular outgrowth of the alar plate part of hp1 with a small preoptic (non-evaginated) contribution from the alar plate of hp2 and is, therefore, not the rostral-most part of the neural tube (Rubenstein et al. 1994; Puelles and Rubenstein 2003; Puelles 2018). The rostral-most region of the neural tube is the acroterminal domain (At), which gives rise to the eye vesicles and several median unpaired structures (Puelles and Rubenstein 2015).

Most of our current understanding of the boundaries of the hypothalamus and its nuclei in rhesus monkeys is derived from comparative architectonic and topographic analyses of sections stained for Nissl, myelin, or acetylcholinesterase in humans, mice, and rats [e.g., Nauta and Haymaker (1969); Bleier (1984); Rempel-Clower and Barbas (1998)]. The developmental origin of hypothalamic nuclei in the adult brain has been described in atlases of chickens and mice with some comparative analyses in guinea pigs, rhesus monkeys, and humans (Puelles et al. 2012, 2019b; Puelles and Rubenstein 2015; Puelles 2019). However, there is no atlas that aligns the developmental framework of the prosomeric model with the position and limits of the hypothalamus and its nuclei in adult primates.

We used the principles of the prosomeric model to create an atlas of the hypothalamus in the adult rhesus monkey based on the likely developmental origin of each hypothalamic nucleus. We first parcellated classically defined hypothalamic nuclei and other architectonic landmarks in images of matched series of sections using various stains. We then performed an analysis of the topology, or relative invariant position, of each hypothalamic nucleus of the adult rhesus monkey in relation to anatomical landmarks already present during patterning of the neural plate and preserved through development into adult life. We assumed homology of these anatomic landmarks across vertebrate species following current usage in the literature and the precisions proposed by the prosomeric model. Using this topological analysis, we then drew boundaries of adult hypothalamic derivatives of alar or basal plate components of hp1, hp2, the acroterminal domain (At) and adjacent neuromeres in two-dimensional (2D) images and maps, rendered in a 3D reconstruction. The result of our systematic reinterpretation of the hypothalamus in the adult rhesus monkey is an atlas with developmental ontologies for each hypothalamic nucleus. This atlas will help predict causal and hodological connections within the hypothalamus and with regard to other CNS structures in primates and disruption in pathological states.

## Methods

### Animal cases, perfusion, and tissue processing

We analyzed sections from 7 young adult rhesus monkeys (*Macaca mulatta*; Table 1). These cases were also used for other studies (Barbas et al. 1993; Dombrowski and Barbas 1996; Zikopoulos and Barbas 2006; Ghashghaei et al. 2007; Garcia-Cabezas et al. 2017). Detailed protocols were approved by Institutional Animal Care and Use Committees (Harvard Medical School and Boston University School of Medicine) according to NIH guidelines [DHEW Publication no. (NIH) 80-22, revised 1996, Bethesda, MD, USA]. Rhesus monkeys were deeply anaesthetized with a lethal dose of sodium pentobarbital (~50 mg/kg, intravenous, to effect) and perfused transcardially, with 4% paraformaldehyde in either cacodylate buffer or PBS (0.1 M, pH 7.4). Brains were removed from the skull, photographed, cryoprotected in ascending sucrose solutions (10–25% in PBS 0.01 M at pH 7.4), frozen in −75 °C isopentane (Fisher Scientific, Pittsburgh, PA, USA) for rapid and uniform freezing (Rosene et al. 1986), and cut in the coronal plane on a freezing microtome (AO Scientific Instruments, Reichert Technologies; Buffalo, NY, USA) at 40 or 50 µm to produce 10 matched series.

**Table 1:** Summary of cases and stains

Case	Myelin	SMI-32	WFA	NADPH/NOS	DARPP-32	AChE	Nissl	PV	CB	CR
AL	+				+		+			
AN		+		+			+			
AP		+					+			
AQ	+			+	+		+			
AT			+			+	+			
AX							+			+
AZ						+	+	+	+	

### Assays and stains

To visualize nuclei and architectonic landmark features of the hypothalamus, we stained series of sections for Nissl, myelin, acetylcholinesterase (AChE), nicotinamide adenine dinucleotide phosphate (NADPH/NOS), wisteria floribunda agglutinin (WFA), dopamine and adenosine 3′-5′monophosphate (cAMP) regulated phosphoprotein of M(r) 32kDA (DARPP-32), non-phosphorylated intermediate microtubule protein SMI-32 (SMI-32), and calcium-binding proteins parvalbumin (PV), calbindin (CB) and calretinin (CR). Assays and stains for each animal case are summarized in Table 1. We stained sections with thionin blue for Nissl as previously described (García-Cabezas et al. 2016; Timbie et al. 2020). We stained myelin using the Gallyas silver technique (Gallyas 1979; Zikopoulos et al. 2016). For AChE staining, we followed the protocol described previously (Wang and Barbas 2018). For NADPH/NOS staining, a marker for localizing nitric oxide synthase (NOS), we followed the protocol of Dombrowski and Barbas (1996). DARPP-32 was stained as described previously (Barbas et al. 1993; Zikopoulos et al. 2016, 2017). Staining for SMI-32 was done as described previously (Medalla and Barbas 2006). Staining for PV, CB, and CR calcium-binding proteins was done as described previously (Dombrowski et al. 2001).

We stained extracellular matrix and perineuronal nets with WFA for antigen retrieval. Briefly, free-floating sections were rinsed in PBS (0.01 M, pH 7.4) and incubated in hydrogen peroxide (0.3% in PBS, Sigma-Aldrich) for 30 min. For antigen retrieval sections were incubated in sodium citrate buffer (0.01 M, pH 8.5, at 80–85 °C) for 30 min. Then, sections were rinsed in glycine (0.05 M in PBS 0.01 M at pH 7.4) for 1 h and pre-blocked [20% bovine serum albumin (BSA) and 0.2% Triton X-100 in PBS 0.01 M at pH 7.4] for 3 h followed by overnight incubation in WFA (Biotinylated Wisteria floribunda lectin, cat. no. B-1355, Vector Laboratories, Burlingame, CA, USA; diluted 1:200 in 20% BSA and 0.2% Triton X-100 in PBS 0.01 M at pH 7.4). We then incubated sections for 1 h in avidin–biotin horseradish peroxidase complex (AB-HRP kit; Vectastain PK-6100 ABC Elite kit, Vector Laboratories; diluted 1:100 in 0.01 M PBS), rinsed in PBS and processed for the peroxidase-catalysed polymerization of diaminobenzidine (DAB; Vector or Zymed Laboratories Inc., South San Francisco, CA, USA; 0.05% DAB and 0.004% H_2_O_2_) for 2–3 min under microscopic control.

For SMI-32 staining, we rinsed sections as described for WFA and pre-blocked them [10% normal goat serum (NGS), 5% BSA and 0.2% Triton X-100 in PBS 0.01 M at pH 7.4]. We then incubated sections overnight with primary antibodies against SMI-32 (Sternberger Monoclonals, Lutherville, MD, USA; diluted 1:5000 in 1% NGS, 1% BSA and 0.1% Triton X-100 in PBS 0.01 M at pH 7.4), rinsed in PBS and incubated for 4 h in the secondary biotinylated antibody (goat anti-mouse IgG, cat. no. BA-9200; Vector Laboratories, diluted 1:200 in PBS, 1% NGS, 1% BSA and 0.1% Triton X100). We then incubated sections for 1 h in avidin–biotin horseradish peroxidase complex (AB-HRP kit; Vectastain PK-6100 ABC Elite kit, Vector Laboratories; diluted 1:100 in 0.01 M PBS), rinsed in PBS and processed for the peroxidase-catalysed polymerization of diaminobenzidine (DAB; Vector or Zymed Laboratories; 0.05% DAB and 0.004% H_2_O_2_) for 2–3 min under microscopic control.

For DARPP-32 staining, we used monoclonal antibodies to purified DARPP-32 (Dr. Hugh Hemmings, Laboratory of Molecular and Cellular Neuroscience, Rockefeller University; gift from Dr. Paul Greengard). To visualize neurons labeled with antibodies to DARPP-32, series of sections were washed (3 × 30 min each in 0.01 M PBS, 0.15 M NaCl at pH 7.4 followed by 10% horse serum) and then briefly washed (5 min) and placed overnight at room temperature in antiserum to DARPP-32 (diluted 1:30,000 in PBS). The next day, sections were removed from primary antiserum and washed 3 times (10 min each in PBS). Sections were incubated for 4 h in the secondary biotinylated antibody (goat anti-mouse IgG; Vector Laboratories, diluted 1:200, in PBS, 1% NGS, 1% BSA, 0.1% Triton-X) and rinsed in PBS. The sections were then processed for immunohistochemistry according to the instructions for the Vector ABC kit (Vector Labs, Burlingame, CA) as described above.

For PV, CB, and CR, antibodies that are specific for the calcium-binding proteins without cross reactivity were purchased from commercial sources (Swiss Antibodies, anti-PV #235, monoclonal; anti-CB D-28k #300, monoclonal; anti-CR #7696, polyclonal). Sections were washed (3 × 10 min each in 0.1 M PBS at pH 7.4) and placed in 6% goat serum for 1 h. Sections were then rinsed (3 × 10 min in 0.1 M PBS), placed in 0.1 M PBS solution containing antiserum for either PV (1:2000 dilution), CB (1:2000 dilution) or CR (1:2000 dilution) and incubated for 72 h at 4 °C with gentle agitation. Sections were then rinsed (3 × 10 min each in 0.1 M PBS). Sections were incubated for 4 h in secondary biotinylated antibody (goat anti-mouse or goat anti-rabbit IgG; Vector Laboratories, diluted 1:200, in PBS, 1% NGS, 1% BSA, 0.1% Triton-X, Vector Laboratories), and rinsed in PBS. The sections were then processed for immunohistochemistry according to the Vector ABC kit (Vector Labs, Burlingame, CA, USA) as described above.

Sections were mounted on gelatin-coated slides and dried, and some were counterstained for Nissl (García-Cabezas et al. 2016), dehydrated in graded alcohols, cleared in xylenes, and coverslipped with mounting media (Permount, Fisher Scientific; or Entellan, EM Sciences, Hatfield, PA, USA).

### Photography for hypothalamic atlas

We captured images of the hypothalamus in sections stained for AChE (Case AZ), Nissl (Case AT), WFA (Case AT), SMI-32 (Case AP), myelin (Case AQ), PV (Case AZ), CB (Case AZ), and CR (Case AX) with an Olympus DP70 camera mounted on an Olympus BX51 microscope at 12.5× magnification and brightfield illumination to create 2D and 3D atlases of the hypothalamus in the adult rhesus monkey. We captured images with the same camera at 200× to visualize perineuronal nets with WFA staining. We captured images with the same camera at 100× for neuron populations with specific calcium-binding proteins. We traced the boundaries of hypothalamic nuclei over images of AChE stained sections using Adobe Illustrator CC software (Adobe Systems Incorporated, San José, CA, USA) and used the same software to assemble figures. We made minor adjustments to overall brightness and contrast but did not retouch images.

For 3D reconstruction, we aligned series of images of the hypothalamus in Nissl stained sections using Reconstruct™ software (version 1.1.0.0) as described (Fiala 2005). We traced outlines of nuclei through aligned Nissl stained sections to generate 3D Boissonat structures. No other adjustments were made.

### Mapping for analysis of calcium-binding protein distribution

We used exhaustive plotting to map the distribution of calretinin-positive (CR+) neurons in representative coronal sections of the hypothalamus (Case AX) as described previously (Joyce and Barbas 2018). Briefly, we used a semi-automated commercial system (Neurolucida, Version 2018.1.1; Olympus BX60 microscope) to plot the architectonic landmarks of the limits of the hypothalamus, including the third ventricle, the optic tract, the optic chiasm, the infundibulum, and the cerebral peduncle, at 40× magnification under brightfield illumination. We then plotted each CR+ labeled neuron in the hypothalamus at 400× magnification using a marker (red) to represent each CR+ neuron. We then compared each map to matched sections of the hypothalamus stained for AChE to trace the limits of hypothalamic nuclei for qualitative analysis of the distribution of CR+ neurons. No other adjustments were made.

## Results

### Rationale for a topological reinterpretation of the hypothalamus in adult rhesus monkeys

What makes the nuclei and anatomical landmarks of the hypothalamus hypothalamic? We first acknowledge, as others have, that the classical topography of this brain region and its nuclei is arbitrary and, at times, problematic (Rempel-Clower and Barbas 1998; Puelles et al. 2012; Puelles 2019). For example, certain terms like “posterior area” and “dorsal area” are anatomically misleading (the majority of the “posterior area” is found dorsal to the “dorsal area” and is not in the posterior-most position of the adult hypothalamus). Further, there are many names used to describe homologous nuclei across vertebrate species, resulting in significant confusion for the study of the hypothalamus and its complex functions. Thus, until recently, it has not been possible to precisely define and study the underlying anatomical structures of the hypothalamus that contribute to its complex functions.

Before the advent of genetic and molecular studies, there was no rationale for naming of the hypothalamus and its constituent nuclei that informed structure, developmental origin and homology across species—a hypothalamic ontology. The prosomeric model proposes a developmental ontology for the constituent nuclei of the hypothalamus by systematic extrapolation of adult hypothalamic structures from fated embryological units based on: (1) genoarchitecture and (2) topology (Puelles and Rubenstein 2015; Puelles 2019). Topology, consistent with its use in mathematics, refers to the invariant position of structures through continuous change. A prosomeric topology, in principle, may be related to the Japanese art of folding paper, origami. Beginning with a planar geometry, folds are introduced to a square piece of paper in a specific sequence and scale that results in a novel figure, like a swan or a frog. If one unfolds the origami figure, one can visualize geometric units of the paper that were shaped to contribute to its final form. As in origami, the organizers present in the early neural plate induce genetic signals that fate topological units to their final position in the adult CNS. A unique genoarchitectural profile for each neuromere, plate, and progenitor area of the neural plate provides a signature pattern for each brain region that is retained throughout secondary and tertiary morphological events in CNS development and into adult architecture. Thus, a prosomeric topology, like a developmental road map, makes it possible to identify and trace hypothalamic landmarks and nuclei to be identified and traced across a developmental sequence from the progenitor area to the final adult position.

Accordingly, we consider as hypothalamic the adult derivatives of the three prosomeres of the secondary prosencephalon, apart from the structures derived from the telencephalic vesicle as outlined in the next section.

### A brief summary of the prosomeric model

We briefly describe the main principles of the prosomeric model in relation to the developing hypothalamus and adjacent brain regions (Rubenstein et al. 1994; Puelles and Rubenstein 2003, 2015; Puelles et al. 2012; Nieuwenhuys and Puelles 2016; Puelles 2018). The prosomeric model proposes a stereotyped process of regionalization (patterning or specification) of the CNS during early development caused by gradients of signaling molecules that spread from organizers (Fig. 1). The floor and roof plates pattern the neural plate along its dorso-ventral axis and specify longitudinal alar and basal plates. Other organizers pattern the neural plate along its rostro-caudal axis into transverse segments, or protosegments [spinal cord (sc), rhombencephalon (Rhomb), mesencephalon (Mes), diencephalon (Dien), and secondary prosencephalon (SPro)]. Each protosegment is further patterned into several neuromeres, each composed of alar and basal plate parts. Thus, the intersection of longitudinal plates and transverse neuromeres forms a checkboard pattern of orthogonal boundaries already present at the neural plate stage (Fig. 1a). This pattern constitutes a fundamental Bauplan for the development of the neural plate which is apparently common to all vertebrates.

The prosomeric model divides the diencephalon into three neuromeres (p1, p2, and p3) and the secondary prosencephalon into three neuromeres [hp1, hp2, and acroterminal domain (At); Fig. 1a–c]. The alar plate part of p2 gives rise to the dorsal thalamus and the alar plate part of p3 gives rise to the prethalamus (classic ventral thalamus), which includes the reticular nucleus (R; formerly the thalamic reticular nucleus, TRN (Puelles and Rubenstein 2015; Nieuwenhuys and Puelles 2016), the zona incerta (ZI), and prethalamic nuclei [PTh; formerly known as the “dorsal” and “posterior” hypothalamic areas (Puelles et al. 2012); Fig. 1d]. The basal plate parts of p2 and p3 contribute a part of the mesodiencephalic substantia nigra (SN) and other dopaminergic cell groups. Further, the prethalamic prosomere (p3) that forms a rostral boundary between the diencephalon and the secondary prosencephalon expands and creates during morphogenesis of this interface a significant topographical and topological discontinuity between the dorsal thalamus (p2) and the caudal-most hypothalamic prosomere (hp1; Fig. 1a–d). The adult hypothalamus is derived from neurogenic units within the secondary prosencephalon and is regionalized dorsoventrally into the alar and basal parts of hp1, hp2, and the acroterminal domain (At; Fig. 1d). The alar plate part of hp1 also gives rise to most of the pallium (Pal) and subpallium (SPal) of the evaginated telencephalic vesicle (Tel), which gives rise to the entire cerebral cortex, plus the basal ganglia, septum, and amygdala formations (Fig. 1d). The preoptic area (POA) is one of four banded regions that comprise the telencephalic subpallium and arises from the alar plate part of hp2 (Fig. 1d). The acroterminal domain (At) functions as an organizer. The alar plate part of the At gives rise to the preoptic terminal lamina, the retina, and the optic chiasm (oc), while the basal plate part of the At gives rise to structures of the prospective neurohypophyseal portal system, including the infundibulum (INF) and neurohypophysis (h). The rostral-most termination of the roof plate is the prospective anterior commissure (ac), which lies topographically dorsal to the POA at the end of development (Fig. 1a–d).

Topologically, the hypothalamus is rostral to p3 and ventral to the telencephalon. Note that at the end of morphogenesis, due to the bending of the longitudinal axis of the neural tube, the hypothalamus topographically lies below (i.e., ventral) to the thalamus (Fig. 1d). The difference between the topological and topographic positions of the hypothalamus is due to the development of the marked cephalic flexure. This positioning is maintained through adulthood and represents a 90° angle change from the early patterned units of the hypothalamus in the neural plate. It follows that a causally meaningful prosomeric ontology applied to the hypothalamus would emphasize naming structures and limits by the relative invariant positioning (topology) of the structures and nuclei patterned during the early neural plate stage. Thus, what classical studies refer to as “ventral” or “dorsal” according to topography are better classified as “rostral” or “caudal” in topological terms within the prosomeric model. In our atlas, we chose to allow more time for a future emergent consensus on axial nomenclature and instead follow the terminological example of classical studies (Nauta and Haymaker 1969; Bleier 1984; Rempel-Clower and Barbas 1998) to refer to the relative topographical location of hypothalamic landmarks and nuclei in adult rhesus monkeys.

We use the color code in Fig. 1 across all figures for consistency to denote the likely developmental origin of hypothalamic nuclei and boundaries in adult rhesus monkeys. We traced adult hypothalamic derivatives of the alar plate in solid blue lines, and derivatives of the basal plate in solid pink lines. Adult derivatives from hp1 are shown in dark shades of pink and blue and those of hp2 are shown in lighter shades. Adult derivatives of p3 are traced in yellow perforated lines.

### Architectonic parcellation and developmental ontology of hypothalamic nuclei in rhesus monkeys

We based the parcellation of classic hypothalamic nuclei in adult rhesus monkeys on images of matched coronal brain sections stained with Nissl and AChE. We further visualized the boundaries of hypothalamic nuclei using additional stains (Table 1). The latter stains confirmed some architectonic boundaries of adult hypothalamic nuclei or landmarks that were already well-defined in Nissl and AChE stained sections. We then divided the hypothalamus of the adult rhesus monkey into three topographic regions (“Caudal”, “Tuberal and Medial”, and “Rostral”) (Table 2) following comparative architectonic analyses in mice, rats, and humans (Rempel-Clower and Barbas 1998). In these three hypothalamic regions, we traced 18 hypothalamic nuclei whose architectonic features are comparable across primate and rodent species, as described previously (Rempel-Clower and Barbas 1998; Puelles et al. 2012). Of note, we included the subthalamic nucleus (STN) in our analysis because modern genoarchitectonic studies have shown that it is a distinct nucleus of the adult hypothalamus (Puelles et al. 2012). Further, we excluded the posterior, dorsal, and anterior hypothalamic areas and the preoptic area because the same studies have shown that they are not part of the hypothalamus proper. Specifically, the anterior hypothalamic area was absorbed into the preoptic area (POA), which is considered one of four regions of the telencephalic subpallium—a secondary derivative of the alar plate of hp2. The posterior and dorsal hypothalamic areas were reassigned as prethalamic nuclei (PTh) because they originate from p3 (Puelles et al. 2012).

**Table 2:** Topographic regions of the rhesus monkey hypothalamus and their constituent nuclei

Region	Nucleus	Abbreviations
Caudal	Lateral mamillary nucleus	LM
	Medial mamillary nucleus	MM
	Paramamillary nucleus	PM
	Perimamillary nucleus	PeM
	Subthalamic nucleus	STN
	Supramamillary nucleus	SM
Tuberal and medial	Area of the tuber cinereum	TCA
	Arcuate nucleus	ARC
	Dorsomedial nucleus	DM
	Perifornical nucleus	Pef
	Premamillary nucleus	PreM
	Tuberomamillary nucleus	TM
	Ventromedial nucleus (core and shell)	VMc/VMs
Rostral	Lateral hypothalamic area	LA
	Paraventricular nucleus (magnocellular and parvicellular groups)	PaM/PaP
	Rostral paraventricular nucleus	RPa
	Suprachiasmatic nucleus	SCH
	Supraoptic nucleus	SO

We thus reinterpreted the classical structure, limits, and nuclei of the adult hypothalamus of rhesus monkeys using a prosomeric ontology. We represented the assumed developmental origin of hypothalamic structures using the same color scheme throughout the figures. Our analysis of the likely developmental origin by progenitor prosomere (hp1, hp2) and plate of origin (alar, basal plates) of 18 parcellated nuclei in the hypothalamus of adult rhesus monkeys is based on homology of the adult mouse in Table 3.

**Table 3:** Comparison of nomenclature of hypothalamic nuclei in the mouse and rhesus monkey

Alar plate				Basal plate		
Progenitor area (mouse)	Adult derivative (mouse)	Adult homologue (rhesus)		Progenitor area (mouse)	Adult derivative (mouse)	Adult homologue (rhesus)
hp1						
DPa	Dorsal entopeduncular n.^a^			RTuD	Magnocellular lateral hypothalamic n.^a^	
CPa	Prereticular lateral hypothalamic area	Lateral hypothalamic area		RTuI	Peduncular dorsomedial n.	Dorsomedial n.^b^
	Pre-eminential lateral hypothalamic area				Ventrobasal perifornical n.	Perifornical n.
	Peduncular supraoptic n.^a^				Tubermamillary n.^b^	Tuberomamillary n.
	Paraventricular n. (magnocellular)	Paraventricular n. (magnocellular)		RTuV	Hypothalamic ventricular organ^a^	
VPa	Paraventricular n. (parvicellular)	Paraventricular n. (parvicellular)		PRM	Posterior hypothalamic n.^a^	
SPa	Ventral entopeduncular n.^a^			RM	Paramamillary n.	Paramamillary n.
	Subparaventricular n.^a^				Supramamillary n.	Supramamillary n.
					Perimamillary n.	Perimamillary n.
					Subthalamic n.	Subthalamic n.
hp2						
TPa	Rostral paraventricular n.	Rostral paraventricular n.		TuD	Anterobasal area	Area of the tuber cinereum
	Terminal supraoptic n.	Supraoptic n.			Ventromedial n (shell)^a,b^	
	Optic ventricular recess			TuI	Median eminence	
	Entrance of optic nerve				Neurohypophyseal diverticule	
TSPa	Anterior hypothalamic n.^a^	Rostral paraventricular n.			Arcuate n.	Arcuate n.
	Optic chiasm				Terminal dorsomedial n.	Dorsomedial n.^b^
	Optic tract				ventromedial n. (core)^b^	Ventromedial n.
	Suprachiasmatic n.	Suprachiasmatic n.		TuV	Tuberomamillary n.^b^	Tuberomamillary n.
					ventral tuberal area^a^	
					Hypothalamic ventricular organ^a^	
				PMp	Dorsal premamillary n.	Premamillary n.
				Mp	Lateral mamillary n.	Lateral mamillary n.
					Medial mamillary n	Medial mamillary n

^a^Molecularly defined nucleus in adult mice without clear definition in stained sections used in this study of the rhesus monkey

^b^Nucleus of basal plate origin with mixed progenitor and migratory pattern from hp1 and hp2 proposed by the prosomeric model (Puelles et al. 2012). Ontological analysis of progenitor area and adult derivatives in mouse were adapted from the same authors

### Topological analysis of hypothalamic boundaries in adult rhesus monkeys

To test this developmental ontology, we conducted an analysis of the topology of architectonic landmarks of the secondary prosencephalon and the diencephalon in relation to adult hypothalamic nuclei. The prosomeric model provides a framework to help explain the development of the entire CNS following a topology, or fated position, which can be traced from the early neural plate to adult positioning. Topological relationships of regions of the CNS persist through development into adulthood with homology in many architectonic landmarks (Rubenstein et al. 1994; Puelles and Rubenstein 2003; Puelles and Ferran 2012; Puelles et al. 2012; Nieuwenhuys and Puelles 2016; Puelles 2018). Thus, we assumed homology and extrapolated to rhesus monkeys the topological relations of landmarks such as longitudinal or transverse tracts, commissures, or decussations that appear in early neuroepithelial stages of developing mice. These landmarks retained a similar relative topographical positioning in relation to hypothalamic nuclei in monkeys as seen in adult mice.

In the caudal hypothalamus, we selected the following anatomical landmarks: the mamillary bodies (Mb), the subthalamic nucleus (STN), and the diencephalic prosomere p3 which contains the reticular nucleus (R), the prethalamic nuclei (PTh), the zona incerta (ZI), part of the substantia nigra (SN) and the internal capsule (ic), whose fibers course longitudinally through p3 but originate elsewhere. In the tuberal and medial hypothalamus, we selected the optic tract (ot), the internal capsule (ic), and p3 derivatives (the PTh, the ZI, and the R). For the rostral hypothalamus, we selected the anterior commissure (ac), the preoptic area (POA), p3 derivatives (the PTh, the ZI, and the R), the longitudinal optic tract (ot), the optic chiasm (oc), and the internal capsule (ic).

**Fig. 2:**
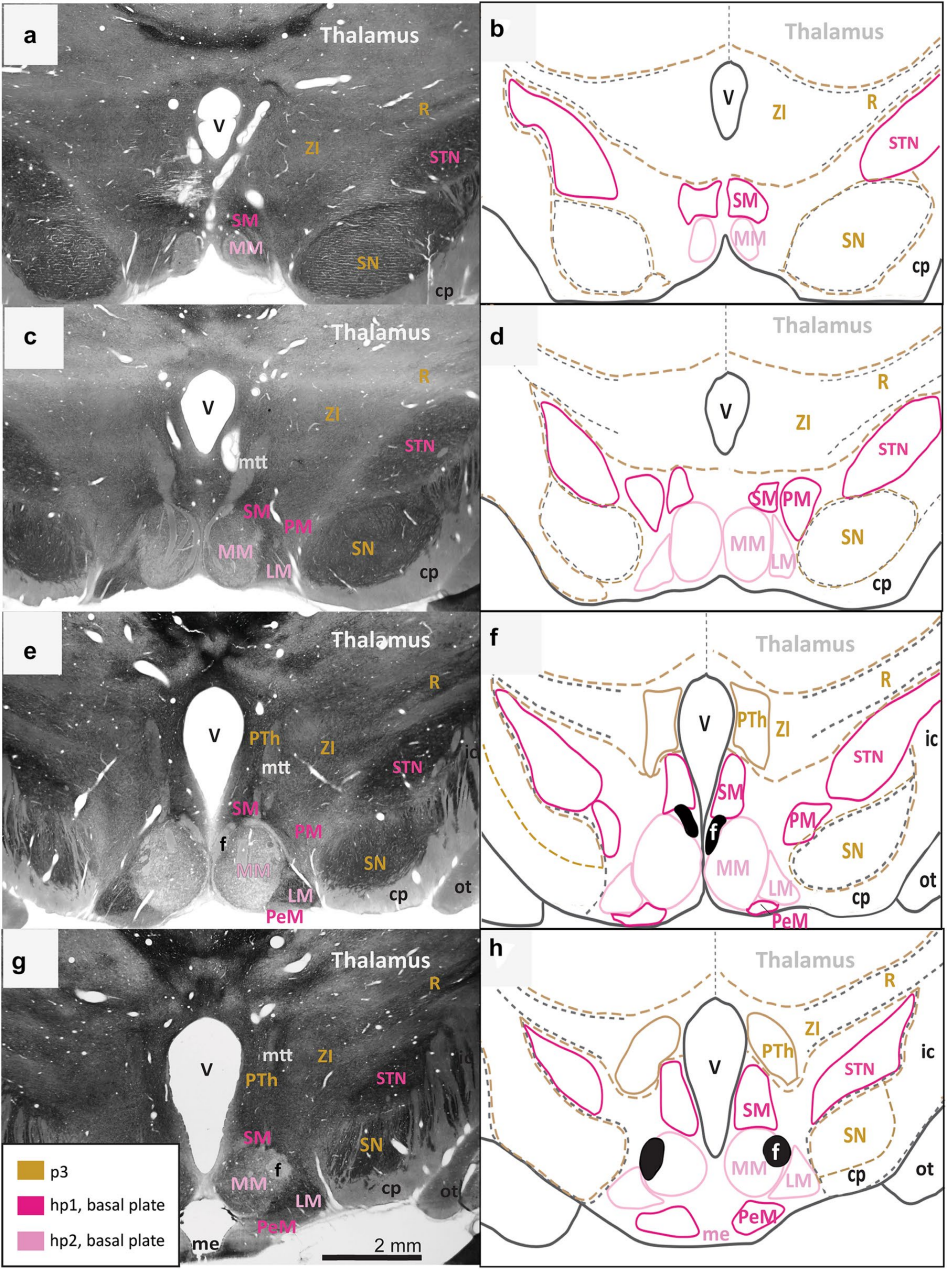
Architectonic boundaries of the caudal hypothalamus in the adult rhesus monkey show developmental origin of anatomical landmarks and hypothalamic nuclei. **a**, **c**, **e**, **g** Photomicrographs of coronal sections through the hypothalamus of an adult rhesus monkey stained for AChE; **b**, **d**, **f**, **h** maps of traced architectonic boundaries. Sections are ordered sequentially from caudal **a**, **b** to rostral **g**, **h**. All nuclei of the caudal hypothalamus are likely of basal plate origin. The SM, the PM, the STN, and the PeM are likely derived from hp1 while the MM and the LM likely arise from hp2. Calibration bar (2 mm) in **g** applies to **a**, **c**, **e**, and **g**. For color code see Fig. 1. See list of abbreviations for complete terms

**Fig. 3:**
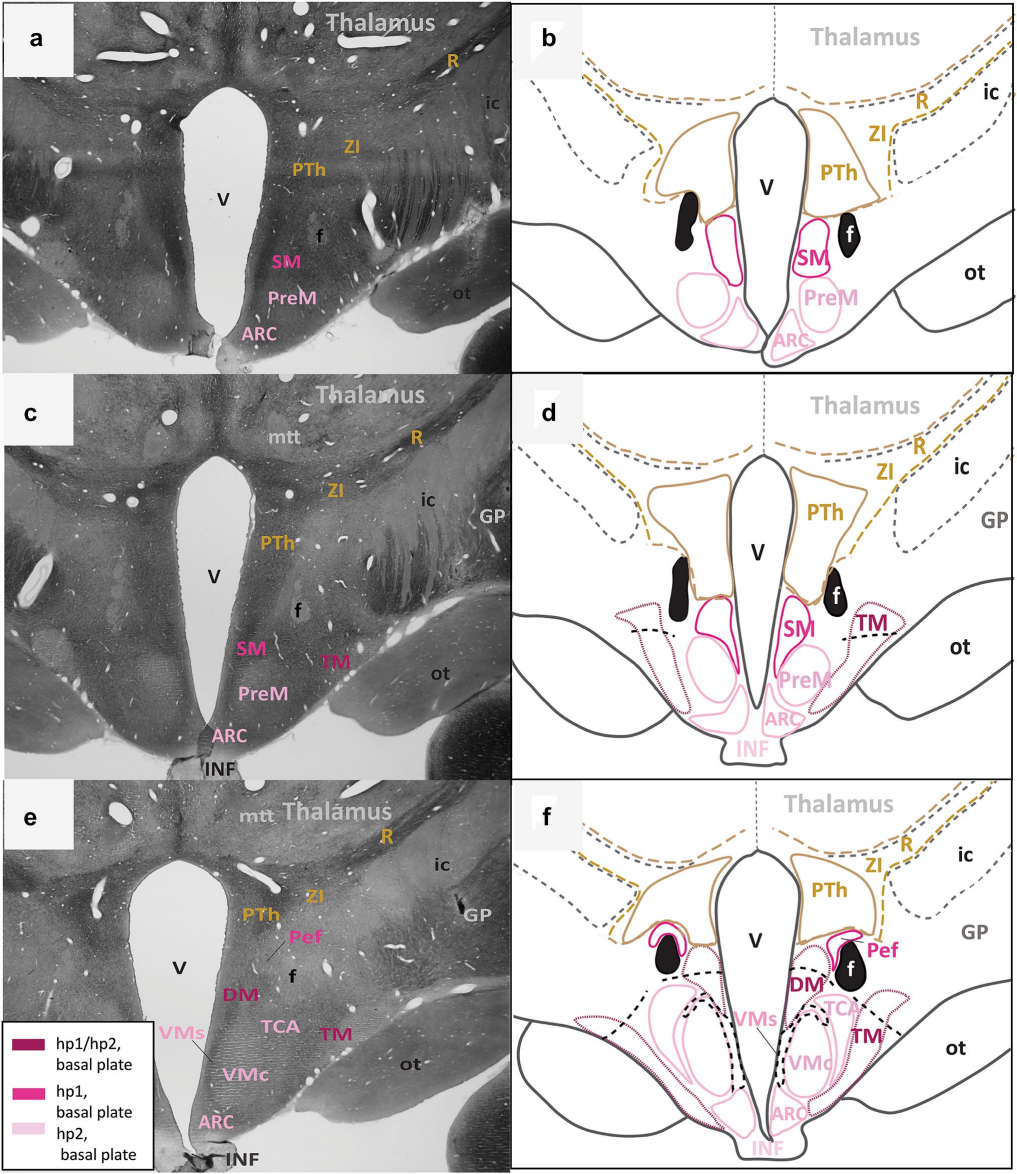
Architectonic boundaries of the tuberal and medial hypothalamus in the adult rhesus monkey show developmental origin of anatomical landmarks and hypothalamic nuclei. **a**, **c**, **e** Photomicrographs of coronal sections through the hypothalamus of an adult rhesus monkey stained for AChE; **b**, **d**, **f** maps of traced architectonic boundaries. Sections are ordered sequentially from caudal **a**, **b** to rostral (**e**, **f**). All nuclei of the tuberal and medial hypothalamus are of basal plate origin. The SM and the Pef are likely derived from hp1 while the PreM, the ARC, the TCA, and the VMc likely arise from hp2. The DM and the TM are thought to be derived from the basal plate parts of both hp1 and hp2 (maroon). Proposed architectonic boundaries of the VMs line the VMc medially to meet the ventral boundary of the DM. Proposed boundaries dividing the DM and the TM into likely hp1 or hp2 parts are also shown. For color code see Fig. 1. See list of abbreviations for complete terms

**Fig. 4:**
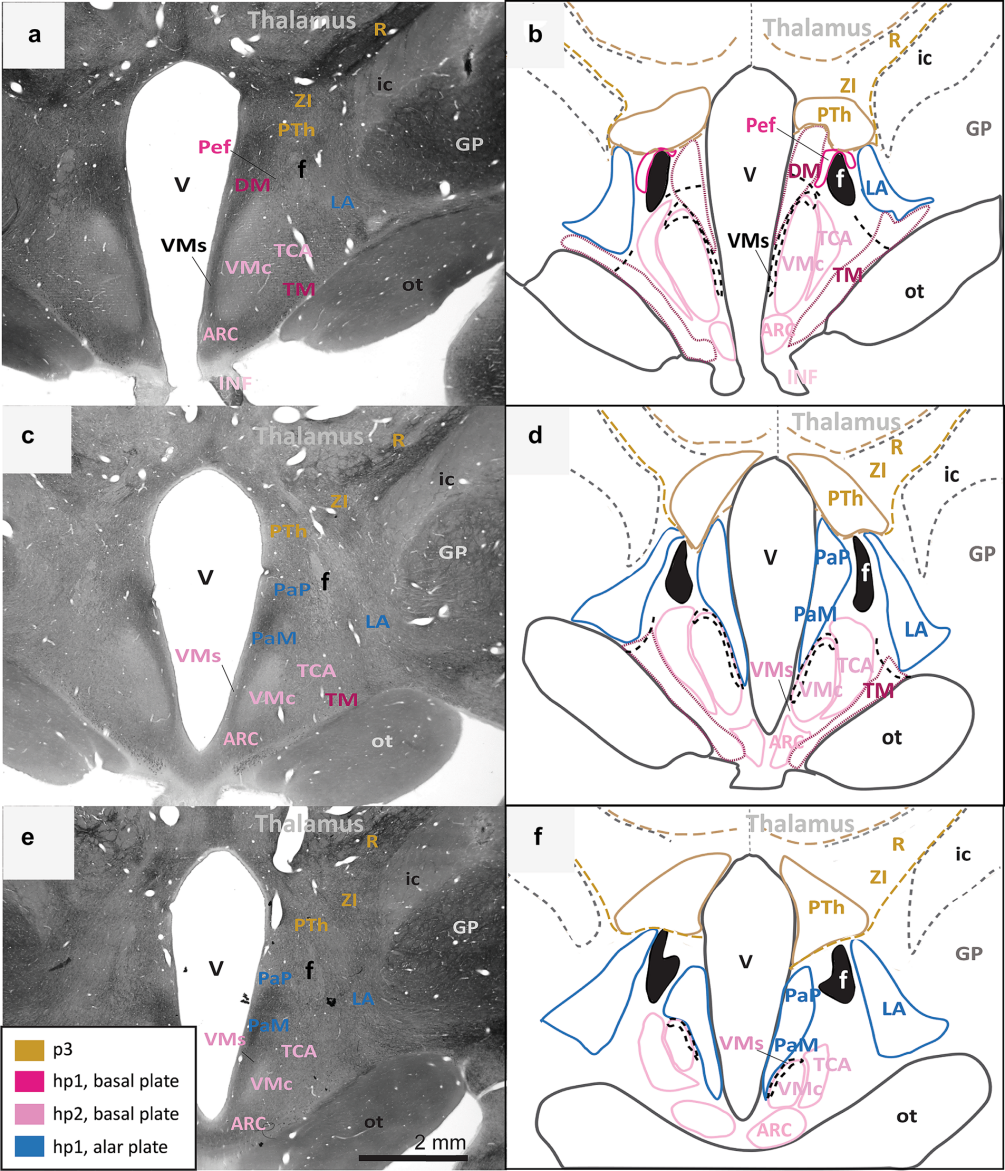
Continuation of the architectonic boundaries of the tuberal and medial hypothalamus in the adult rhesus monkey show developmental origin of anatomical landmarks and hypothalamic nuclei. **a**, **c**, **e** Photomicrographs of coronal sections through the hypothalamus of an adult rhesus monkey stained for AChE; **b**, **d**, **f** maps of traced architectonic boundaries. Sections are ordered sequentially from caudal **a**, **b** to rostral (**e**, **f**). All nuclei of the tuberal and medial hypothalamus are of basal plate origin. All nuclei of the rostral hypothalamus are of alar plate origin. The Pef is the rostral-most nucleus of the basal plate part of hp1. The Arc, the TCA, and the VMc arise from the basal plate part of hp2. The DM and the TM are thought to be derived from basal plate parts of both hp1 and hp2 (maroon). Proposed architectonic boundary of the VMs lines the VMc medially and courses dorsally to meet the DM. The LA, the PaM, and the PaP are likely derived from the alar plate part of hp1. Calibration bar (2 mm) in e applies to **a**, **c**, **e**. For color code see Fig. 1. See list of abbreviations for complete terms

**Fig. 5:**
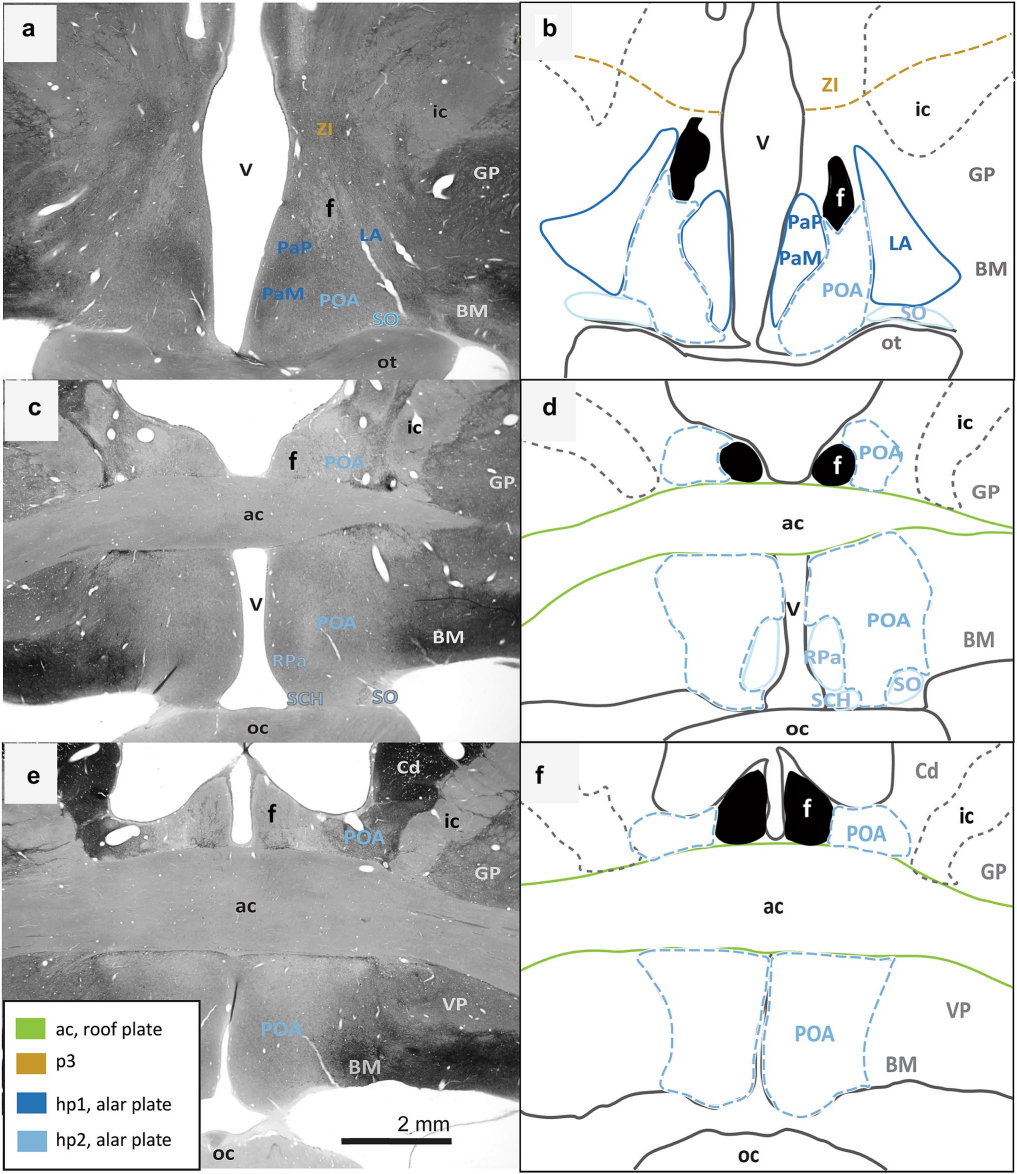
Architectonic boundaries of the rostral hypothalamus in the adult rhesus monkey show developmental origin of anatomical landmarks and hypothalamic nuclei. **a**, **c**, **e** Photomicrographs of coronal sections through the hypothalamus of an adult rhesus monkey stained for AChE; **b**, **d**, **f** maps of traced architectonic boundaries. Sections are ordered sequentially from caudal **a**, **b** to rostral (**e**, **f**). All nuclei of the rostral hypothalamus are of alar plate origin. The LA, the PaM, and the PaP are derived from hp1 while the RPa, the SO, and the SCH arise from hp2. The POA is excluded from the hypothalamus in the adult rhesus monkey according to Puelles et al. (2012). Calibration bar (2 mm) in e applies to **a**, **c**, **e**. For color code see Fig. 1. See list of abbreviations for complete terms

We first identified and traced the adult derivatives of p3 in gold and the other landmarks in black (Figs. 2, 3, 4, 5). We then searched systematically for interneuromeric boundaries as follows: between adjacent neuromeres of the diencephalon and secondary prosencephalon (p3-hp1); between hypothalamic prosomeres (hp1, hp2); between the topologically rostral-most hypothalamic prosomere and the acroterminal domain (hp2-At); between the hypothalamic part of the rostral-most hypothalamic prosomere and the preoptic area (hp2-POA); and an intrahypothalamic boundary between the alar and basal plates (ABb).

**Fig. 6:**
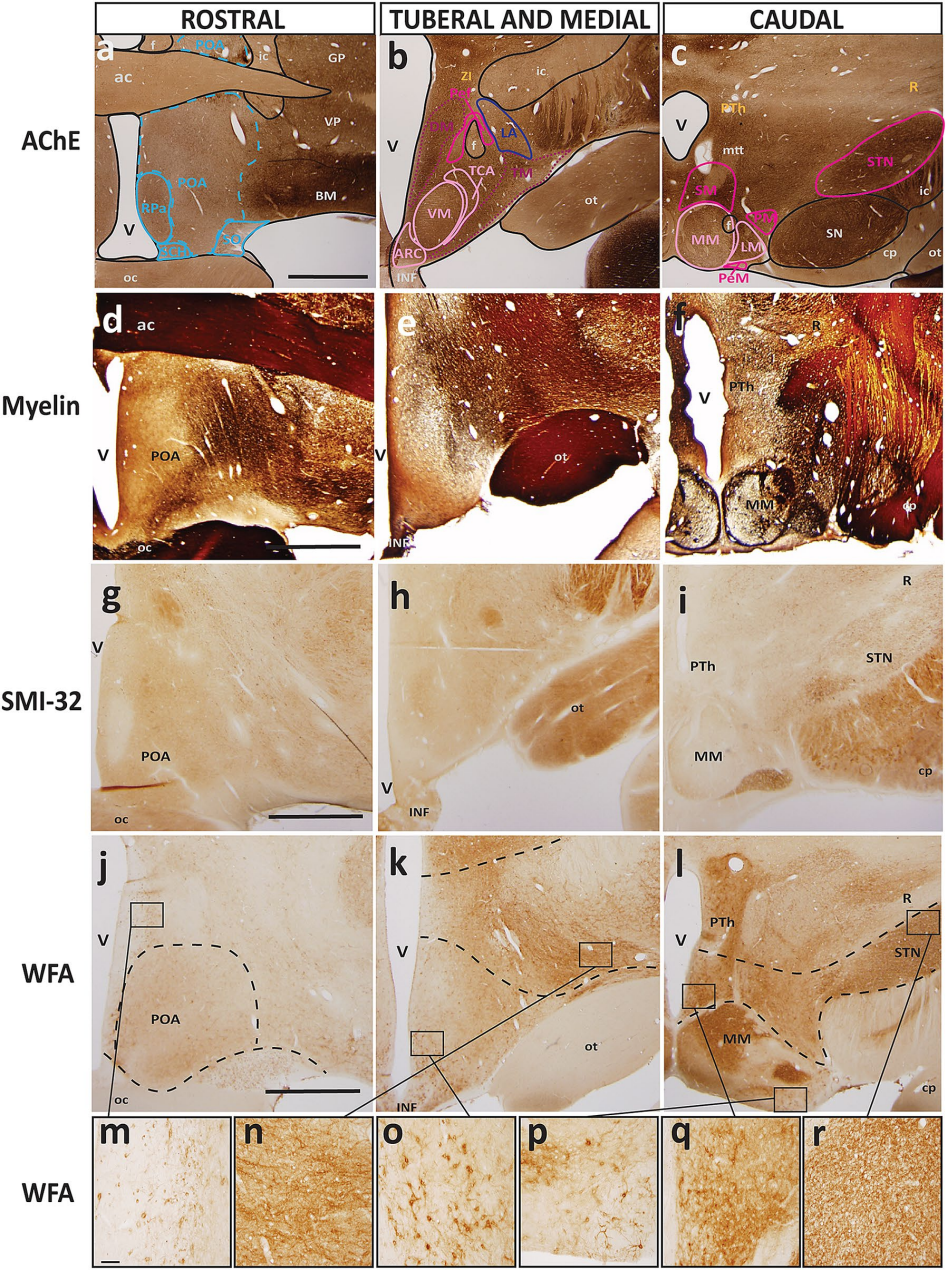
Representative sections of each topographic region of the adult hypothalamus visualized with distinct stains. **a**–**c** Traced maps of hypothalamic nuclei by prosomere and plate of origin from representative sections from each of the three topographical regions of the hypothalamus of rhesus monkey visualized with acetylcholinesterase (AChE; case AZ). Matched sections were stained for: **d**–**f** myelin (case AQ); **g**–**i** SMI-32 (case AP); **j**–**l** WFA (case AT). WFA was the only stain used that clarified interneuromeric boundaries between p3, hp1 and hp2 (drawn in dashed lines). We visualized medium and large neurons with WFA in the: **m** magnocellular group of the paraventricular complex (PaM); **n** lateral hypothalamic area (LA); **o** arcuate nucleus (ARC); **p** perimamillary nucleus (PeM); **q** supramamillary nucleus (SM); **r** subthalamic nucleus (STN). Calibration bar (2 mm) in sections **a**, **d**, **g**, **j** apply to all sections within corresponding rows at low magnification. Calibration bar (50 µm) in section **m** applies to all sections **m**–**r** at higher magnification. For color code in traced maps see Fig. 1. See list of abbreviations for complete terms

First, we assessed the interneuromeric boundary between adult derivatives of p3 and hp1 (p3-hp1). We also traced an interneuromeric boundary between the derivatives of two sequential diencephalic neuromeres (p2, p3) that are found topologically caudal to the derivatives of hp1 (Figs. 2a, 3, 4, 5f). The p2-p3 (zona limitans) boundary is shown from the limits of the caudal hypothalamus and lies dorsal to the anterior commissure (ac; Fig. 5c–f). We then traced boundaries along derivatives of p3 (the R, the ZI, and the PTh) medially to the third ventricle (V; Figs. 2a, 3, 4, 5f). We also followed the internal capsule (ic) through the hypothalamus to the cerebral peduncle (cp) and the basal plate part of p3 that contains components of the mesodiencephalic substantia nigra (SN; Fig. 2e–h). The topographically ventral boundary of p3 derivatives is continuous with the dorsal limits of both hp1b derived nuclei (Figs. 2a, 3, 4b) and hp1a derived nuclei (Figs. 4a, 5d). Of note, we visualized a clear neuromeric band of derivatives of p3 using the alternative stain wisteria floribunda agglutinin (WFA; Fig. 6j–l), which further clarified the p3-hp1 boundary, as described below.

Then we analyzed an interneuromeric boundary between the adult derivatives of hp1 and hp2 (hp1-hp2). While we were unable to visualize an hp1-hp2 boundary using AChE and Nissl staining, we saw a potential hp1-hp2 boundary using WFA (see next section; Fig. 6j–l). Additionally, the fornix (f) courses from the rostral (dorsal by topology) to the caudal (ventral by topology) hypothalamus almost exclusively through hp1 and close to the hp1-hp2 boundary, as previously [(Puelles and Rubenstein 2015); Figs. 2, 3, 4, 5]. A branch of the fornix diverges rostrally from the basal plate part of hp1 into the MM in hp1 (Fig. 2). Thus, from the rostral hypothalamus to the MM, the fornix progressively descends with a topographic relation among hp1 derivatives and inserts into the MM. According to prosomeric topology, nuclei in the adult hypothalamus that are dorsal to the fornix are likely derived from hp1 while those ventral to the fornix are likely derived from hp2.

Next, we analyzed the boundary between the adult derivatives of hp2 and the acroterminal domain (hp2-At). The infundibulum (INF), an adult remnant of the basal plate part of the acroterminal domain (along with part of the arcuate nucleus, median eminence, and hypophyseal formation), lines the ventral border of the adult derivatives of hp2b (Figs. 3c, 4d). As the infundibulum disappears under the emergence of the optic tract (ot), another derivative of the acroterminal domain (At) signals a progressive transition from basal plate to alar plate derivatives of the acroterminal domain (At) and hp2 (Fig. 4a–f).

We then analyzed the rostral (topographically dorsal) boundary between adult derivatives of hp2 and the preoptic area (hp2-POA). A portion of the optic tract (ot) extends from the topographically ventral optic chiasm (oc) and courses within the hypothalamus along nuclei derived from the alar plate part of hp2. Where the optic tract meets the optic chiasm, the POA emerges medially between the likely adult derivatives of hp2a (Fig. 5c–d). Thus, the POA contacts the rostral terminus of hp2a and expands dorsally and rostrally to meet the anterior commissure (ac; Fig. 5e–f).

We found neither intrahypothalamic nor adjacent landmarks in the adult rhesus monkey that clearly delineate a boundary between the alar and basal plates (ABb; in rodents, the approximate topological ventral border of the optic tract) through the derivatives of the secondary prosencephalon and in coronal sections alone. Only the infundibulum and the optic tract differentially line the likely adult hypothalamic derivatives of the alar and basal parts of hp2. Figure 4 shows a progressive but gradual caudo-rostral transition between nuclei likely derived from the basal plate to those likely derived from the alar plate. Further, there are no adult hypothalamic nuclei with a mixed migratory pattern across the alar and basal plates according to genoarchitectonic studies (Puelles et al. 2012; Ferran et al. 2015). Thus, while the alar-basal boundary cannot be visualized in adult primates with available methods, the relative invariant position (topology) of derivatives of the alar and basal plate is retained.

### Analysis of alternative stains for resolution of hypothalamic boundaries

In addition to AChE and Nissl, we analyzed coronal brain sections that were stained to demonstrate architectonic properties of the adult hypothalamus and its constituent nuclei. These stains included Gallyas silver stain for myelin, nicotinamide adenine dinucleotide phosphate (NADPH/NOS) for a subclass of interneurons, wisteria floribunda agglutinin (WFA) for extracellular matrix and perineuronal nets, dopamine- and cyclic-AMP-regulated phosphoprotein of molecular weight 32,000 (DARPP-32), and non-phosphorylated intermediate neurofilament protein SMI-32. Figure 6 shows representative sections of the three topographic hypothalamic regions with these stains with matched sections in AChE and traced maps demonstrating architectonic limits of nuclei by prosomere and plate of origin. We use the same color code in Fig. 6 as in Figs. 2, 3, 4, 5.

Some of the alternative stains confirmed the limits of more prominent nuclei, such as the subthalamic nucleus (STN) or suprachiasmatic nucleus (SCH), that are also visualized readily in AChE and Nissl stains. Of note, we confirmed previous observations that the hypothalamus is largely devoid of myelin (Nauta and Haymaker 1969), but we add to this a list of other common stains that seem to leave the hypothalamus entirely untouched yet consistently show major fiber tracts and/or surrounding subcortical structures. We were unable to visualize any of the nuclei of the hypothalamus with myelin, NADPH/NOS, DARPP-32, and SMI-32 stains. Regarding structures close to hypothalamic nuclei, we visualized the prominent capsule of myelin that surrounds the medial mamillary nucleus (MM) and the subthalamic nucleus (STN) to a similar extent. Major fiber tracts, such as the optic tract and the fornix, which course through the hypothalamus, were visualized in all the alternative stains. The appearance of these fiber tracts in each stain was comparable to their appearance in AChE and Nissl. However, the architectonic properties of adult hypothalamic nuclei were not clearly elucidated using these staining techniques, demanding alternative methods of study.

Of note, we visualized the extracellular matrix and perineuronal nets of medium and large neurons with WFA that fell within the boundaries of several adult hypothalamic nuclei derived from the alar and basal plate parts of hp1 (Fig. 6j–l). These included the magnocellular group of the paraventricular complex (PaM; Fig. 6m), the lateral hypothalamic area (LA; Fig. 6n), the subthalamic (STN; Fig. 6r) perimamillary (PeM; Fig. 6p) and supramamillary nuclei (SM; Fig. 6q). We also visualized a clear neuromeric boundary between the derivatives of the basal plate parts of p3 and hp1, and a second boundary between the derivatives of the basal plate parts of hp1 and hp2 with WFA staining (Fig. 6l). In contrast, we found many large neurons within the boundaries of the arcuate nucleus (ARC; Fig. 6o), which is thought to be a derivative of the basal plate part of hp2 with some neurons contributed by the acroterminal domain (At). This was the only nucleus derived from hp2 with perineuronal net staining that clearly framed the soma of neurons as well as primary dendrites. This population of stained neurons in the ARC extended ventrally into the median eminence but were not found within the limits of the ventromedial nucleus (VM).

### Analysis of calcium-binding proteins for resolution of hypothalamic boundaries

**Fig. 7:**
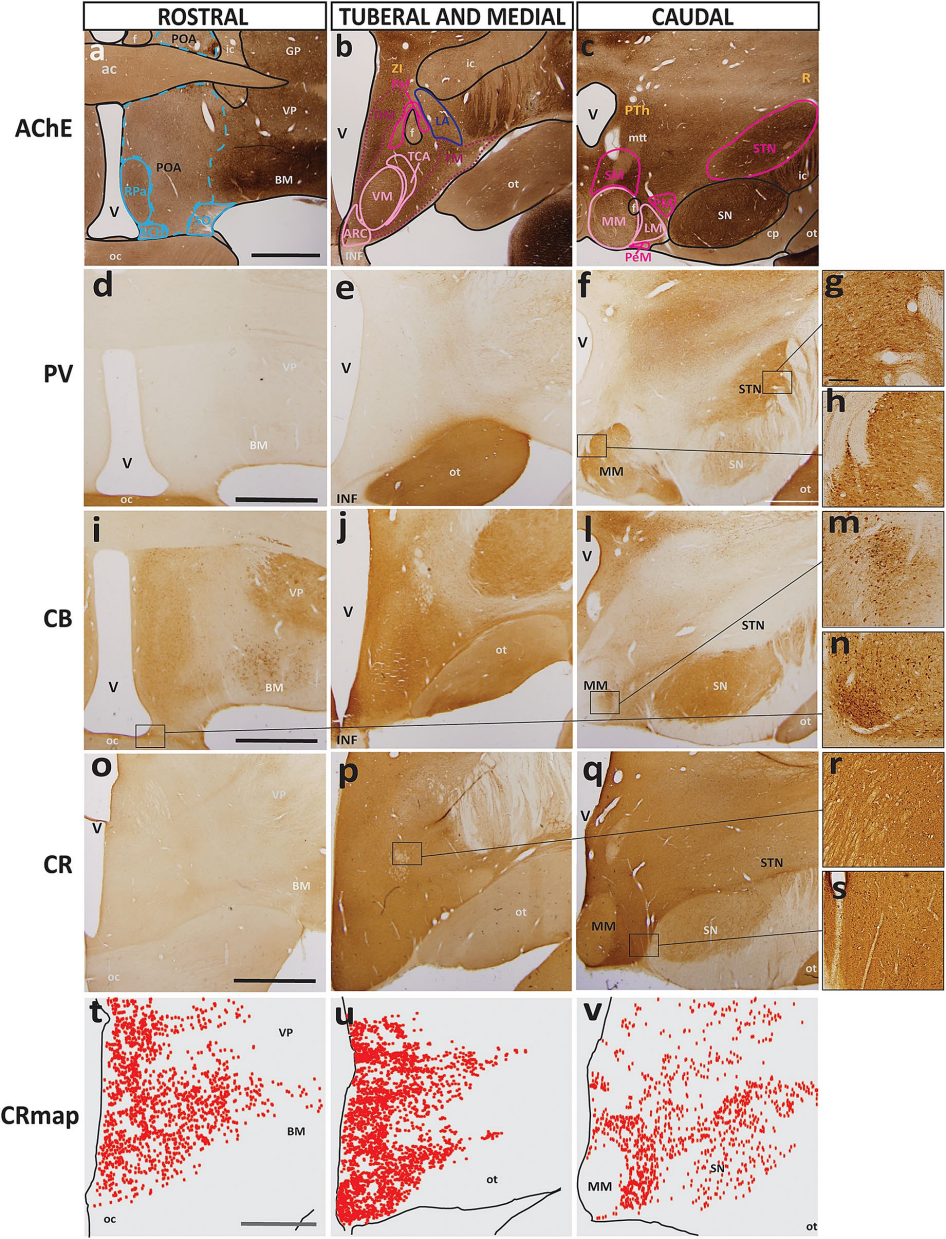
Representative sections of each topographic region of the adult hypothalamus visualized with AChE or stains for calcium-binding proteins. **a**–**c** Traced maps of hypothalamic nuclei by prosomere and plate of origin from representative coronal sections from each of the three topographical regions of the hypothalamus of rhesus monkey stained for acetylcholinesterase (AChE; case AZ). Representative matched sections from each topographical region of the hypothalamus of adult rhesus monkey stained for the calcium-binding proteins: **d**–**f** parvalbumin (PV; case AZ), **i**–**l** calbindin (CB; case AZ), **o**–**q** calretinin (CR; case AX). **t**–**v** Maps of exhaustive plotting of CR neurons (CR map) in representative sections show the distribution and relative density of CR when compared to PV and CB in the hypothalamus. Large PV+ neurons were found in: **g** STN; and **h** MM. In stark contrast, large CB+ neurons were only found in: **m** MM; and **n** SCH. **r**, **s** Small CR+ neurons were only found dispersed between hypothalamic nuclei with a semi-uniform distribution. Calibration bar (2 mm) in sections **a**, **d**, **i**, **o**, **t** apply to all sections within corresponding rows at low magnification. Calibration bar (200 µm) in **g** applies to sections **g**, **h**, **m**, **n**, **r**, **s** at higher magnification. For color code in traced maps see Fig. 1. See list of abbreviations for complete terms

We analyzed sections stained for parvalbumin (PV), calbindin (CB), and calretinin (CR) to visualize the distribution of neurons with different calcium-binding proteins. These proteins label subclasses of interneurons in the prefrontal cortex of the rhesus monkey (Dombrowski et al. 2001) but also excitatory projection neurons in the dorsal thalamus (Jones 2007). Figure 7 shows representative sections of each of the three topographic regions of the hypothalamus stained for neurons with calcium-binding proteins and a traced map of nuclei by prosomere and plate of origin over sections stained with AChE for visual comparison.

The distribution of parvalbumin-positive (PV+) and calbindin-positive (CB+) neurons was sparse compared to the distribution of calretinin-positive (CR+) neurons in the adult hypothalamus. A few PV+ neurons were found in the medial mamillary nucleus (MM; Fig. 7h) near the origin of the mamillothalamic tract (mtt) as well as in the lateral part of the subthalamic nucleus (STN; Fig. 7g). There were no other PV+ neurons found in the hypothalamus, and PV only gave a visual contrast (without specifically staining neurons) to the optic tract (ot) and some portions of p3, including the reticular nucleus (R). In stark contrast, and without specifically staining neurons, calbindin (CB) seemed to localize in structures in a way that mirrored that of PV. Sparse collections of CB+ neurons were found within several hypothalamic nuclei, including the medial mamillary (MM; Fig. 7m), lateral mamillary (LM), suprachiasmatic (SCH; Fig. 7i), and arcuate (ARC) nuclei and the lateral hypothalamic area (LA). There were also some small CB+ neurons sporadically found in the areas between all nuclei and within the preoptic area (POA).

On the other hand, PV+ and CB+ neurons were far outnumbered by CR+ neurons (Fig. 7o–q). CR+ neurons were small but had a much higher density by comparison with the larger but sparsely distributed PV+ and CB+ neurons given the same sample area. Thus, we used images of stained sections as well as exhaustive mapping to visualize the distribution of calretinin-positive (CR+) neurons in the adult hypothalamus (Fig. 7t–v). The distribution of CR+ neurons in the adult hypothalamus was extensive, forming a diffuse matrix of small neurons found both within and between the limits of nearly all hypothalamic nuclei (Fig. 7t–v). We were unable to confirm if these neurons were also PV+ and/or CB+ neurons. By qualitative comparison, it is unlikely that CR+ neurons are also PV+ neurons due to the stark difference in appearance of stained sections, but it is plausible that small CB+ neurons may also be CR+ as the two cases had some overlap in stained neuron distribution. There was a noted absence of CR+ neurons in the medial mamillary (MM; Fig. 7v), supraoptic (SO; Fig. 7t) and suprachiasmatic (SCH; Fig. 7t) nuclei, which are all derivatives of hp2 and the acroterminal domain (At).

### Reconstruction of adult hypothalamic nuclei in 3D

**Fig. 8:**
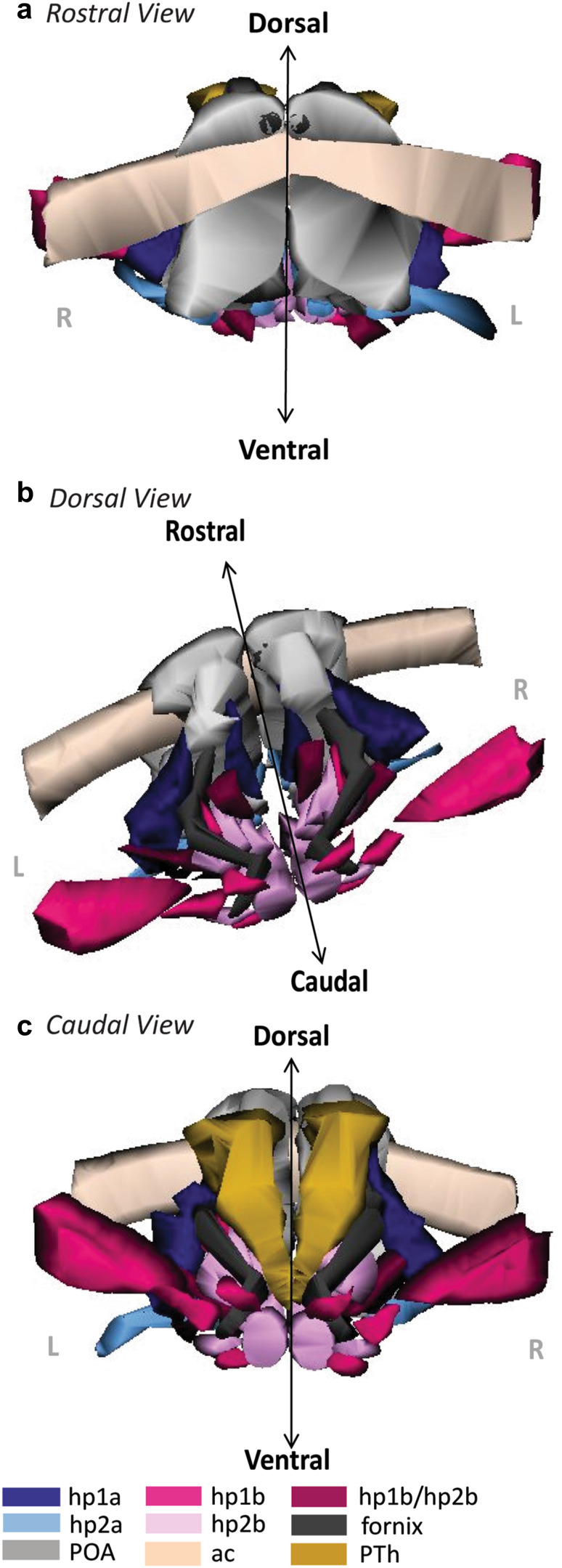
3D reconstruction of the hypothalamus of the adult rhesus monkey shows developmental origin of anatomical landmarks and nuclei. **a** Rostral view of the adult hypothalamus bound by the anterior commissure (ac, beige) and preoptic area (POA, grey). **b** Oblique-dorsal view looking down into the third ventricle with the anterior commissure, the POA, and the relative spatial positions of alar and basal plate derivatives of hp1 and hp2 nuclei. The fornix (f, black) courses exclusively through alar and basal plate parts of hp1 until it diverges caudally into the MM. **c** Caudal view of the hypothalamus including prethalamic nuclei (PTh) of the diencephalon. The proposed boundaries of the VMs are not shown as it could not be visualized in any stain used in our analysis. For color code see Fig. 1. See list of abbreviations for complete terms

**Fig. 9:**
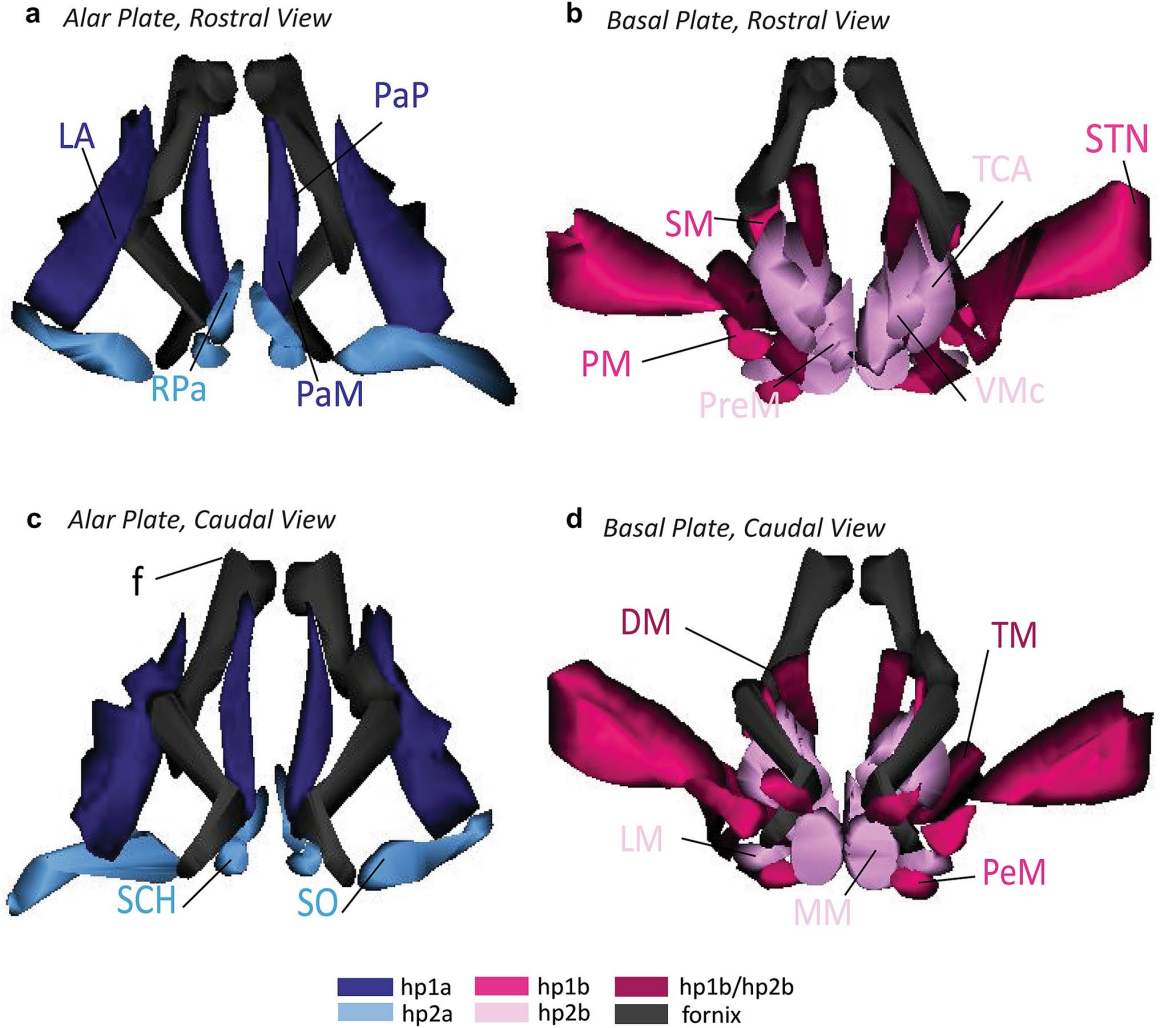
3D reconstruction of the hypothalamus of the adult rhesus monkey shows developmental origin of nuclei in relation to the fornix. **a**, **b** Rostral view of adult hypothalamic nuclei derived from alar **a** and basal **b** plate parts of hp1 and hp2 in the rhesus monkey. **c**, **d** Caudal view of adult hypothalamic nuclei derived from alar **c** and basal **d** plate parts of hp1 and hp2. The proposed boundary of the VMs is not shown as it could not be visualized in any stain used in our analysis. The fornix (f) courses exclusively through alar and basal plate parts of hp1 until it diverges caudally into the MM. For color code see Fig. 1. See list of abbreviations for complete terms

For a novel view of the developmental ontology of the adult hypothalamus, we aligned tracings of each parcellated nucleus with the fornix, the anterior commissure, the POA, the optic tract, and the PTh from Nissl images and reconstructed the tracings in 3D (Figs. 8, 9). The colors used in Figs. 8 and 9 are used across all figures. This reconstruction of the adult hypothalamus provided a visual summary of the ontology and topology of each hypothalamic nucleus in its adult positioning. The 3D model demonstrated the spatial relations between nuclei of different hypothalamic prosomeres and plates of origin while illustrating consistently the principles of the prosomeric model. Further, the topological relations of the adult hypothalamus as seen in 3D helped to visualize the hp2-POA boundary in greater resolution. We could further visualize the boundaries of the PTh and the course of the fornix as landmarks related to nuclei derived from the alar and basal plate parts of hp1 (Fig. 9). Unfortunately, a lateral and dorsal aspect of the hypothalamus did not offer a better view of a potential alar-basal boundary (ABb) without significantly altering the 3D model. Our 3D reconstruction complements the 2D images and maps to understand the spatial relations of adult hypothalamic nuclei by prosomere and plate of origin.

## Discussion

**Fig. 10:**
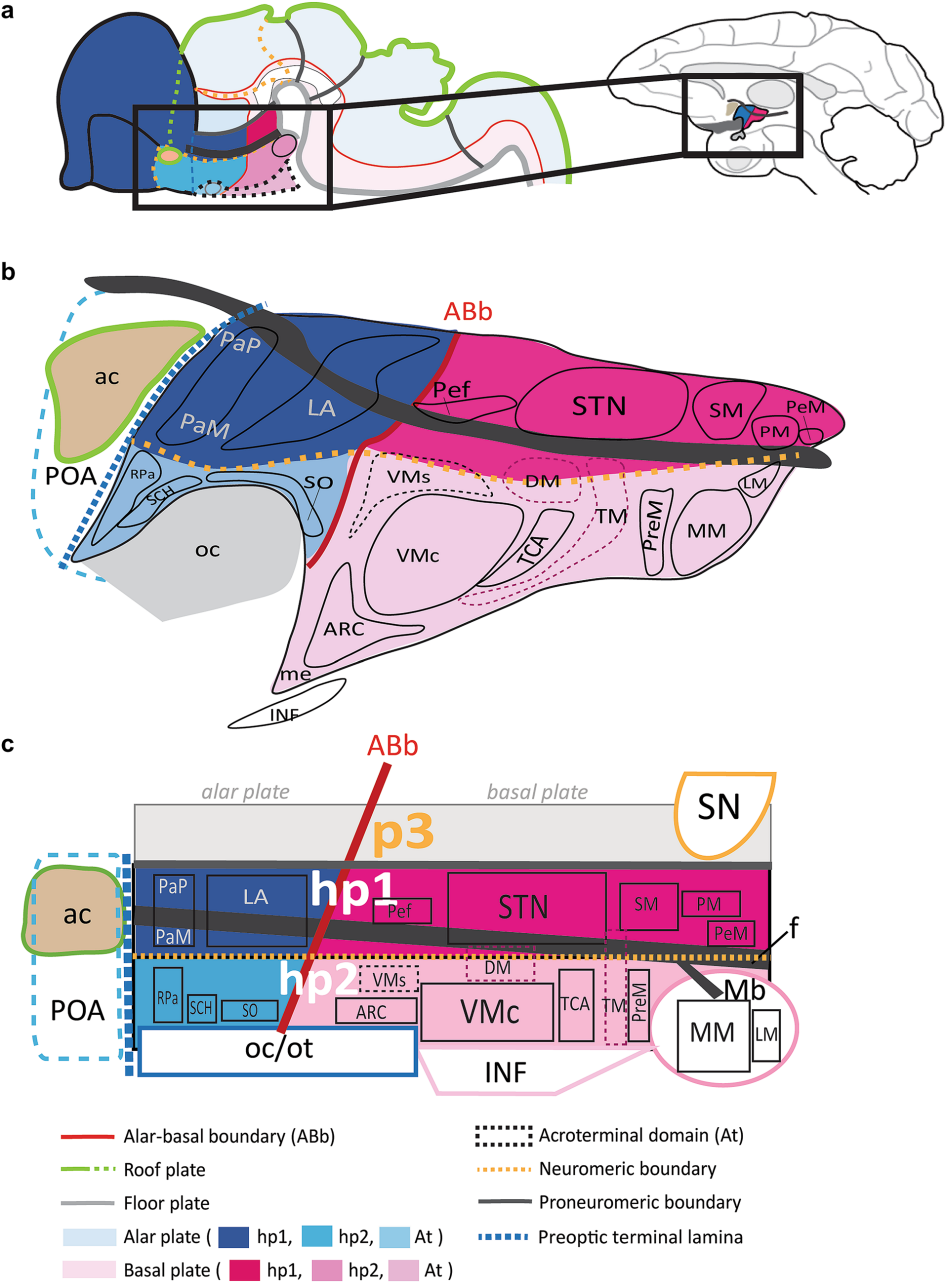
Schematic summary of architectonic boundaries and hypothalamic nuclei in the adult rhesus monkey by likely prosomere and plate of origin. **a** Developmental origin of adult hypothalamic nuclei in the rhesus monkey as predicted by the prosomeric model (Puelles et al. 2012; Puelles and Rubenstein 2015). Architectonic boundaries of the adult hypothalamus in the rhesus monkey are illustrated by relative invariant position (topology) in relation to each hypothalamic prosomere [hp1, hp2, acroterminal domain (At)] and plate of origin (alar and basal plate). Theoretical boundaries of adult hypothalamic nuclei are traced over their likely prosomere(s) and plate of origin. **b** Sketch of architectonic landmarks and nuclear boundaries of the adult hypothalamus in rhesus monkeys with corresponding developmental origin as predicted by the prosomeric model. **c** Technical schematic of architectonic landmarks and nuclear parcellation of the hypothalamus in the adult rhesus monkey by developmental origin. Proposed placement of uncertain boundaries or mixed migratory patterns from both hp1 and hp2 are shown in a dashed line. For color code see Fig. 1. See list of abbreviations for complete terms

The prosomeric model provides a novel framework that explains the patterning and development of the central nervous system as a Bauplan across vertebrates (Rubenstein et al. 1994; Puelles and Rubenstein 2003; Lauter et al. 2013; Dominguez et al. 2014, 2015; Nieuwenhuys and Puelles 2016; Puelles 2018; Schredelseker and Driever 2020). We applied the principles of the prosomeric model to a topological analysis of architectonic landmarks to trace adult hypothalamic nuclei to their likely developmental origin in rhesus monkeys, as summarized in Fig. 10. This analysis relied on tracing distinct hypothalamic nuclei to their respective progenitor plate and neuromere of origin by assuming homology across species, and guided by prominent homologous architectural landmarks (Puelles et al. 2012). This analysis rendered a visual summary of likely adult hypothalamic derivatives in a 2D and 3D atlas.

### Prosomeric topology differs from classical topography

Our analysis of prosomeric topology of the hypothalamus resulted in key differences with some aspects of classical parcellations in the rhesus monkey [e.g., Nauta and Haymaker (1969)]. First, some nuclei included in our atlas were not included in the hypothalamus traditionally (Puelles et al. 2012; Ferran et al. 2015; Puelles and Rubenstein 2015). For example, the subthalamic nucleus (STN) was classically defined as a component of the ventral thalamus in the rhesus monkey (Whittier and Mettler 1949), but new genetic and molecular data have ascribed this nucleus to the hypothalamus (Puelles et al. 2012). Its developmental origin and unique “wing” positioning, as described in other vertebrates (Jiao et al. 2000), and connections with the basal ganglia suggest it may have a distinct functional role from other basal plate nuclei of hp1 (Hashimoto et al. 2003; Puelles et al. 2012; Haynes and Haber 2013). Similarly, the “anterior hypothalamic area” was reassigned to the preoptic area (POA) in accord with the prosomeric model (Puelles et al. 2012), and by its separation from the hypothalamus in rhesus monkeys (Bleier 1984; Rempel-Clower and Barbas 1998). The exclusion of the POA from the hypothalamus proper is further supported by molecular data for inclusion instead with the telencephalic subpallium (Puelles and Rubenstein 2015; Puelles et al. 2016).

On the other hand, the classical parcellation in mice and primates of dorsal and rostral boundaries of the adult hypothalamus was blurred by three classically defined “areas” (Bleier 1984; Rempel-Clower and Barbas 1998; Puelles et al. 2012). Nauta included the dorsal and posterior areas to the hypothalamus to give topographical meaning to a space between a gross dorsal boundary, the hypothalamic sulcus, and prominent nuclei of the tuberal and medial hypothalamus (Nauta and Haymaker 1969). With recent genetic and molecular data, the dorsal and posterior hypothalamic areas are now regarded as “prethalamic nuclei” (PTh) derived from neuromere p3 (Puelles et al. 2012). As shown here, the stain WFA established a more refined dorsal boundary between p3 and the nuclei of the alar and basal parts of hp1 in adult rhesus monkeys. Some PTh neurons likely contribute to the mesodiencephalic dopaminergic system with other p3, p2, p1, m1, and m2 derived structures in rhesus monkeys (Sánchez-González et al. 2005; García-Cabezas et al. 2009). The reassignment of the PTh to p3 may thus connect topological and physiological evidence to distinguish adult diencephalic derivatives from hypothalamo-telencephalic derivatives.

### Some tuberal and medial hypothalamic nuclei have complex developmental origins

The tuberomamillary nucleus (TM), the core and shell parts of the ventromedial nucleus (VMc/VMs), and the dorsomedial nucleus (DM) presented non-trivial challenges to tracing topological boundaries. Developmental evidence in mice has shown complex migratory patterns from progenitor sites to the adult positioning for these three nuclei (Puelles et al. 2012). Architectonic descriptions of the TM in humans, rats, and rhesus monkeys contain discrepancies when defining one or more nuclear substructures, suggesting that the TM may exist as a complex (Rempel-Clower and Barbas 1998). Similarly, at least three distinct nuclei likely make up the VMc while a fourth forms the VMs in mouse (Puelles et al. 2012; Puelles and Rubenstein 2015). While the architectonic boundary of the VMc is prominent in adult rhesus monkeys, we were unable to visualize a VMs with architectonic methods but relied on topological boundaries based on its likely homologue in adult mice. However, it is possible that in primates the VMs follows a different migratory pattern than the VMc or is even absorbed within the boundaries of VMc (Puelles et al. 2012). Similarly, recent genoarchitectural data suggest that the DM is also a nuclear complex from basal hp1 and hp2 (Puelles et al. 2012). We could thus propose topological boundaries for the VMs, the DM, and the TM with hp1-hp2 boundaries, but could not visualize topological boundaries for constituent nuclei of the VMc.

### Alternative stains may indicate plasticity in adult hypothalamus

The architecture of prominent hypothalamic nuclei in monkeys was previously studied with a variety of stains [reviewed in (Rempel-Clower and Barbas 1998)]. The addition of stains beyond AChE and Nissl provided little additional assistance in our hypothalamic parcellation (Figs. 6, 7). Besides the noted exception of WFA, we found that calretinin (CR) stained broadly small neurons within and between nuclei. The relative paucity of label in the hypothalamus with other markers stands in contrast to the surrounding non-hypothalamic structures and tracts, including the globus pallidus, ventral pallidum, thalamus, or reticular nucleus.

The relative lack of staining of the hypothalamus may highlight similarities between the adult hypothalamus and the developing brain for plasticity over a lifetime (Ramón y Cajal 1904a, b; Nauta and Haymaker 1969). The hypothalamus lacks architectonic characteristics traditionally understood to reflect structural stability of different brain regions (De Luca and Papa 2016, 2017; Garcia-Cabezas et al. 2017, 2019), including WFA (Mueller et al. 2016; Bozzelli et al. 2018), SMI-32 (Louis et al. 2012), and myelin (Nauta and Haymaker 1969; Garcia-Cabezas et al. 2019). The paucity of myelin in the hypothalamus suggests retained plasticity throughout life, as suggested by the distribution of markers associated with the plasticity-stability continuum in the prefrontal cortex (Garcia-Cabezas et al. 2017). This pattern was first described by Ramón y Cajal (1904b) in recently born cats and rats, who hypothesized that the relative paucity of myelinated axons in the postnatal hypothalamus, likely meant that the structure and connections of the hypothalamus were in flux much later than the surrounding myelinated brain regions. We provide the adult corollary to Cajal’s hypothesis, demonstrating that the hypothalamus is also largely devoid of myelin in adult rhesus monkeys.

### Anatomic and functional hypothalamic pathways may vary by prosomere and plate of origin

Can the prosomeric model provide a causal and topologically consistent explanation for patterns in adult neural circuitry of the primate hypothalamus? Pathways that connect the hypothalamus with the prefrontal cortex are associated with high cognitive functions including working memory and emotional regulation (Schwartz and Goldman-Rakic 1990; Barbas 1993, 2007; van Eerdenburg and Rakic 1994; Barbas et al. 2018). We previously described a systematic relationship in the connections between prefrontal cortices and the hypothalamus (Rempel-Clower and Barbas 1998). The phylogenetically ancient limbic prefrontal cortical areas in the anterior cingulate and posterior orbitofrontal cortex have the strongest and uniquely bidirectional connections with the hypothalamus (Rempel-Clower and Barbas 1998). Moreover, the pattern and strength of hypothalamic projections varies systematically from the least differentiated limbic areas to the most laminated (eulaminate) prefrontal areas (Rempel-Clower and Barbas 1998), consistent with the rules of the structural model (Barbas and Rempel-Clower 1997), which has been traced to the development and evolution of the cortex (Dombrowski et al. 2001; Barbas 2015; Barbas and García-Cabezas 2016; Garcia-Cabezas et al. 2019; Garcia-Cabezas and Zikopoulos 2019). A recent study also predicted the common developmental origin of connected pallial and subpallial brain regions across mammalian species (Medina et al. 2019). This evidence provides a clear convergence of the prosomeric model and the structural model to predict that the specificity of cortico-hypothalamic connections has a common developmental sequence that likely begins with the specification of the hypothalamus.

A prosomeric topology may also predict specific patterns of connections between the prefrontal cortex and the hypothalamus that conform to the functional compartmentalization of the hypothalamus. Derivatives of the alar plate part of hp1 seem to have a central role in propagating autonomic responses to a variety of external stimuli directly from the prefrontal cortex to the hypothalamus, and from there to the brainstem and spinal cord (Barbas et al. 2003). On the other hand, periventricular cells from the alar plate part of hp1 (paraventricular complex) and hp2 (rostral paraventricular nucleus) origin seem to have an exclusive role in detecting pH changes in cerebrospinal fluid for respiratory and metabolic regulation (Williams et al. 2007; Jalalvand et al. 2018). In contrast, we hypothesize that the likely adult hypothalamic derivatives of the basal part of hp2 seem to have a greater role in neuroendocrine signaling. The exclusive proximity of the arcuate nucleus (ARC) and the core part of the ventromedial nucleus (VMc) to the infundibulum (INF) is likely indicative of a common structural pathway for hypothalamo-pituitary signaling in adult rhesus monkeys.

### Patterning of normal and pathological prosencephalon development

For over a century, the columnar model was assumed to reflect the development of the diencephalon and hypothalamus. The columnar model positioned the hypothalamus as the ventral-most part of the diencephalon and placed the telencephalon as the rostral-most vesicle of the neural tube [Herrick (1910); Kuhlenbeck (1973); reviewed in Puelles and Rubenstein (2015); Puelles (2019)]. Mounting genoarchitectonic evidence strongly supports a new paradigm of CNS development, where progressive regionalization positions the hypothalamus as the antecedent to all pallial and subpallial structures (Puelles et al. 2012; Puelles and Rubenstein 2015). The reassignment of the hypothalamus from the diencephalon to the secondary prosencephalon is centered around a change in the axial terminus of the early neural plate that respects the curve of the cephalic flexure (Puelles et al. 1987). Accordingly, the new axial terminus, termed the acroterminal domain (At), is the rostral-most prosomere of the secondary prosencephalon (Puelles et al. 2012; Puelles and Rubenstein 2015). Thus, the prosomeric model shows that development of pallial and subpallial structures that contribute to the telencephalon are patterned exclusively by the prosomeres of the secondary prosencephalon, i.e., the hypothalamus (Puelles 2011, 2019; Puelles and Rubenstein 2015; Puelles et al. 2019a).

What happens when chromosomal aberrations or teratogenic factors disrupt the patterning of the secondary prosencephalon early in human development? Prosencephalic malformations accompany disruptions to the developmental sequence of the mediolateral patterning of the rostral neural plate (Simon et al. 2000; Solomon et al. 2010; Puelles et al. 2012; ten Donkelaar et al. 2014). A specific subset of malformations known as holoprosencephaly describes a spectrum of interhemispheric cleavage malformations of the forebrain ranging from impaired to complete absence of cleavage. The etiology of holoprosencephaly can be traced in part to specific abnormalities in the Shh (sonic hedgehog) signaling pathway in the early tissues of the rostral neural plate (reviewed in Hong and Krauss 2018; Andreu-Cervera et al. 2019). Consequently, there are massive patterning defects in the hypothalamus and later-appearing structures, including the telencephalon, the eyes, the infundibulum, and the neurohypophysis portion of the pituitary gland. The secondary prosencephalon and its derivatives are thus severely impacted by early genetic aberrations in holoprosencephaly while diencephalic derivatives like the thalamus remain largely unaffected (Simon et al. 2000; Puelles et al. 2012; ten Donkelaar et al. 2014).

The prosomeric model thus explains several aspects of both normal and abnormal patterning of CNS development in ways that the columnar model cannot (Puelles et al. 2012; Puelles and Rubenstein 2015; Nieuwenhuys and Puelles 2016). Given this key developmental finding, we advance the principles of the prosomeric model to examine the relationship of the adult hypothalamus with other regions of the CNS. A better understanding of neurogenesis and neural patterning of the hypothalamus may allow for new strategies to unify the etiology of diseases involving hypothalamic circuits (Radad et al. 2017). The hypothalamus is implicated in neurodegenerative diseases like Alzheimer’s and Parkinson’s (Goldman-Rakic 1987a, b; Ishii and Iadecola 2015; Radad et al. 2017). Symptoms include sleep, metabolic, and non-cognitive abnormalities like neuroendocrine function as potential driver pathologies (Ishii and Iadecola 2015; Radad et al. 2017; Van Erum et al. 2018). Neuraxial pathways from the hypothalamus to the pituitary and adrenal glands also play central roles in dysregulated stress signaling seen in depression, anxiety, and migraine headaches (Min et al. 2014; Myers et al. 2014; Bao and Swaab 2019; Johnson et al. 2019; Kandasamy et al. 2019; Chen et al. 2019). Recent studies in metabolic and hormonal regulation by the hypothalamus seem to support this idea (Grove et al. 2003; Myers et al. 2014; Ishii and Iadecola 2015). These pathologies may represent disruptions to critical regulatory centers for neuraxial pathways of a common developmental origin. A prosomeric topology for hypothalamic nuclei thus appears to predict and explain the roles of hypothalamic nuclei in both normal and pathological signaling pathways.

A modern understanding of the complex structure and connections of the hypothalamus may be elucidated by study of its systematic development. At the nexus of embryological and adult findings likely exists the essence of what makes an adult hypothalamus truly “hypothalamic” and thus integral to structural and neuraxial pathways. We anticipate that our atlas will serve as a valuable tool to study cortico-subcortical pathways involving the hypothalamus and its intricate connectivity with other areas of the CNS. Using the prosomeric model to analyze physiological and pathological pathways may yield clarity and consistency of hypothalamic structure–function relationships. Evaluating patterns in adult pathologies by their impact on prosomeric hypothalamic derivatives may reveal an etiology of these diseases based on changes that may occur at a common time in development that affect networks of anatomic and functional connections.

## Appendix: Box 1

### Definitions of main concepts, theories and principles used in the text

***Bauplan*** a body plan common to all members of the same phylum or subphylum of animals.

***Genoarchitecture*** structural analysis obtained by the study of differential gene expression patterns; applies to embryos and adults.

***Protosegment*** an early transverse (caudo-rostral) segment of the neural tube subsequently subdivided into smaller neuromeres.

***Neuromere*** a transverse unit of the early neural plate specified by caudo-rostral patterning of the neural tube; several neuromeres contribute to a protosegment; in the secondary prosencephalon, these are called prosomeres.

***Plate*** a longitudinal division of the early neural plate specified by the dorso-ventral patterning of the neural tube (i.e., roof, floor, alar, and basal plates).

***Progenitor unit*** a subdivision of the early neural plate patterned by the intersection of longitudinal plates and transverse segments with a characteristic molecular profile; polyclonal neuroepithelial cell populations that share a molecular profile and have homogeneous proliferative and neurogenetic properties.

***Diencephalon*** the protosegment composed of three neuromeres (p1, p2, p3) between the mesencephalon and the secondary prosencephalon that gives rise to adult brain structures such as the dorsal thalamus and the reticular nucleus.

***Secondary prosencephalon ***the rostral-most protosegment of the early neural plate composed of three *prosomeres* (hp1, hp2, At) that gives rise to the entire hypothalamus, the infundibulum, the neurohypophysis, the optic tract, the retina and the telencephalic vesicle.

***Telencephalon*** the rostral vesicle of the alar plate part of the secondary prosencephalon (hp1, hp2) that gives rise to the entire cerebral cortex.

***Nucleus*** a collection of neuronal cell bodies in the adult hypothalamus typically derived from a common *progenitor unit* with a fixed set histogenetic properties.

### Theories and principles

***Columnar model*** a classical model assumed for the development of the diencephalon and telencephalon; positions the telencephalon as the rostral-most termination of the neural plate and includes the hypothalamus in the diencephalon with the epithalamus and the thalamus (Herrick 1910; Kuhlenbeck 1973; Swanson 1992, 2003).

***Prosomeric model*** a predictive model that explains the development of the entire central nervous system; positions the hypothalamus apart from the diencephalon and within the secondary prosencephalon, the rostral-most termination of the neural plate and tube which expands to give rise to the telencephalic vesicle and the underlying hypothalamus, which also carries the eye vesicle (Rubenstein et al. 1994; Puelles and Rubenstein 2003; Nieuwenhuys and Puelles 2016; Puelles 2018).

***Structural model*** a predictive model that relates the pattern and strength of cortico-cortical and cortico-subcortical connections to the graded variation in laminar definition of cortical areas (Barbas and Rempel-Clower 1997; Rempel-Clower and Barbas 1998).

***Topography*** the study of the observed arrangement of the physical features of an area relative to external landmarks (e.g., a set of stereotactic references).

***Topology*** the study of the relative invariant position and spatial relations of areas referred only to the intrinsic neighborhood relationships of its parts among themselves unaffected by continuous processes of change.

## Abbreviations

ABb: Alar-basal boundary
ac: Anterior commissure
ARC: Arcuate nucleus
At: Acroterminal domain
BM: Basal nucleus of Meynert
CB: Calbindin
Cd: Caudate nucleus
CPa: Central paraventricular progenitor area
CNS: Central nervous system
CR: Calretinin
Dien: Diencephalon
DM: Dorsomedial nucleus
DPa: Dorsal paraventricular progenitor area
f: Fornix
GP: Globus pallidus, ventral part
h: Hypophysis
hp1: Hypothalamic prosomere 1
hp1a: Hypothalamic prosomere 1, alar plate part
hp1b: Hypothalamic prosomere 1, basal plate part
hp2: Hypothalamic prosomere 2
hp2a: Hypothalamic prosomere 2, alar plate part
hp2b: Hypothalamic prosomere 2, basal plate part
ic: Internal capsule
INF: Infundibulum
LA: Lateral hypothalamic area
LM: Lateral mamillary nucleus
m1: Mesencephalic neuromere 1
m2: Mesencephalic neuromere 2
Mb: Mamillary bodies
Mes: Mesencephalon
MM: Medial mamillary nucleus
Mp: Mamillary progenitor area
Mtt: Mamillothalamic tract
oc: Optic chiasm
Olf: Olfactory bulb
ot: Optic tract
p1: Diencephalic neuromere 1
p2: Diencephalic neuromere 2
p3: Diencephalic neuromere 3
Pal: Pallium
PaM: Paraventricular complex, magnocellular group
PaP: Paraventricular complex, parvicellular group
Pef: Perifornical nucleus
PeM: Perimamillary nucleus
PreM: Premamillary nucleus
PM: Paramamillary nucleus
PMp: Premamillary progenitor area
PRM: Preretromamillary progenitor area
POA: Preoptic area
Pth: Prethalamic nuclei
PV: Parvalbumin
R: Reticular nucleus
Rhomb: Rhombencephalon
RM: Retromamillary progenitor area
RPa: Rostral paraventricular nucleus
RTuD: Dorsal retrotuberal progenitor area
RTuI: Intermediate retrotuberal progenitor area
RTuV: Ventral retrotuberal progenitor area
sc: Spinal cord
SCH: Suprachiasmatic nucleus
SM: Supramamillary area
SN: Substantia nigra reticulata
SNc: Substantia nigra compacta
SO: Supraoptic nucleus
SPa: Subparaventricular progenitor area
SPal: Subpallium
SPro: Secondary prosencephalon
STN: Subthalamic nucleus
TCA: Area of the tuber cinereum
Tel: Telencephalon
TM: Tuberomamillary nucleus
TPa: Terminal paraventricular progenitor area
TSPa: Terminal subparaventricular progenitor area
TuD: Dorsal tuberal progenitor area
TuI: Intermediate tuberal progenitor area
TuV: Ventral tuberal progenitor area
V: Third ventricle
VMc: Core part of the ventromedial nucleus
VMs: Shell part of the ventromedial nucleus
VP: Ventral pallidum
VPa: Ventral paraventricular area
